# Role of serotype and virulence determinants of *Streptococcus pyogenes* biofilm bacteria in internalization and persistence in epithelial cells *in vitro*

**DOI:** 10.3389/fcimb.2023.1146431

**Published:** 2023-05-10

**Authors:** Feiruz Alamiri, Oscar André, Supradipta De, Pontus Nordenfelt, Anders P. Hakansson

**Affiliations:** ^1^ Division of Experimental Infection Medicine, Department of Translational Medicine, Lund University, Malmö, Sweden; ^2^ Division of Infection Medicine, Department of Clinical Sciences, Lund University, Lund, Sweden

**Keywords:** adherence, epithelial cells, internalization, localization, *Streptococcus pyogenes*, persistence, uptake pathways, virulence factors

## Abstract

*Streptococcus pyogenes* causes a multitude of local and systemic infections, the most common being pharyngitis in children. Recurrent pharyngeal infections are common and are thought to be due to the re-emergence of intracellular GAS upon completion of antibiotic treatment. The role of colonizing biofilm bacteria in this process is not fully clear. Here, live respiratory epithelial cells were inoculated with broth-grown or biofilm bacteria of different M-types, as well as with isogenic mutants lacking common virulence factors. All M-types tested adhered to and were internalized into epithelial cells. Interestingly, internalization and persistence of planktonic bacteria varied significantly between strains, whereas biofilm bacteria were internalized in similar and higher numbers, and all strains persisted beyond 44 hours, showing a more homogenous phenotype. The M3 protein, but not the M1 or M5 proteins, was required for optimal uptake and persistence of both planktonic and biofilm bacteria inside cells. Moreover, the high expression of capsule and SLO inhibited cellular uptake and capsule expression was required for intracellular survival. Streptolysin S was required for optimal uptake and persistence of M3 planktonic bacteria, whereas SpeB improved intracellular survival of biofilm bacteria. Microscopy of internalized bacteria showed that planktonic bacteria were internalized in lower numbers as individual or small clumps of bacteria in the cytoplasm, whereas GAS biofilm bacteria displayed a pattern of perinuclear localization of bacterial aggregates that affected actin structure. Using inhibitors targeting cellular uptake pathways, we confirmed that planktonic GAS mainly uses a clathrin-mediated uptake pathway that also required actin and dynamin. Clathrin was not involved in biofilm internalization, but internalization required actin rearrangement and PI3 kinase activity, possibly suggesting macropinocytosis. Together these results provide a better understanding of the potential mechanisms of uptake and survival of various phenotypes of GAS bacteria relevant for colonization and recurrent infection.

## Introduction

*Streptococcus pyogenes* (Group A Streptococci; GAS) is a versatile human pathogen that causes infections of multiple organ systems (Efstratiou and Lamagni, 2022), with pharyngitis (or “strep throat”) being the most common infection that results in up to 600 million episodes worldwide every year (Carapetis et al., 2005; Tan et al., 2014). Other infections caused by GAS include skin (impetigo, cellulitis, fasciitis) or respiratory tract (otitis media and pneumonia) infections and GAS are also associated with post-infectious sequalae, such as rheumatic fever and glomerulonephritis (Efstratiou and Lamagni, 2022). There are >200 serotypes of GAS (Lancefield, 1962; McMillan et al., 2013; Sanderson-Smith et al., 2014), classified by the surface-exposed M protein, and an individual is often colonized or infected with more than one serotype during their lifetime (Marshall et al., 2015). Niche-specificity is associated with serotype groups so that strains causing pharyngitis (groups A-C) do not commonly cause skin infections (group D) and vice versa, although some serotypes show no specific tissue tropism (group E) (Wannamaker, 1970; Anthony et al., 1976; McMillan et al., 2013; Sanderson-Smith et al., 2014). For pharyngitis, recurrent infections with the same serotype are common in children and in severe cases require removal of the tonsils through tonsillectomy (Ogawa et al., 2011; Dan et al., 2019).

Adherence to respiratory tract cells or injured skin surfaces are initial steps taken by this pathogen to colonize and potentially facilitate their subsequent multiplication and invasion of local tissue, where they can cause infection (Muotiala et al., 1997; Brouwer et al., 2016). However, colonization does not always result in infection and asymptomatic carriage of GAS due to micro-aggregation of bacteria as well as biofilm formation on mucosal surfaces is common (DeMuri and Wald, 2014; DeMuri et al., 2018). At least 20%, and probably more, of children colonized with GAS in the oropharynx do not exhibit any symptoms (Roberts et al., 2012). When infection occurs, the treatment of choice is penicillin or amoxicillin, as beta-lactam resistance is, so far, extremely rare (Vannice et al., 2020). Beta-lactams do not efficiently eliminate bacteria inside eukaryotic cells, therefore any recurrence of pharyngeal infection is thought to be due to the release and growth of GAS residing inside cells upon termination of treatment. Several studies have shown that GAS enter and survive in low numbers inside cells. A higher uptake and prolonged intracellular persistence of biofilm bacteria as compared to planktonic bacteria has been observed by us and others, however the mechanisms involved are, so far, not completely clear (Ogawa et al., 2011; Marks et al., 2014).

Persistent colonization requires the expression of various bacterial factors, such as M protein and hyaluronic acid capsule, that are associated with adherence and persistence in the host niche (Ashbaugh et al., 2000; Courtney et al., 2002; Nobbs et al., 2009). Capsule has been suggested to increase and promote biofilm formation (Matysik and Kline, 2019) and M protein has also been suggested to be involved in biofilm formation through increasing bacterial surface hydrophobicity and adherence to various surfaces (Lembke et al., 2006; Courtney et al., 2009). Surprisingly, in a recent study, we were unable to detect a direct role of M protein or capsule during GAS biofilm formation on prefixed epithelial cells, whereas expression of Streptolysin S (SLS) and cysteine protease SpeB impaired this process (Alamiri et al., 2020). Therefore, although their role in biofilm formation has been elucidated, the role of these virulence factors during internalization and persistence of biofilm bacteria have not been studied.

In epithelial cells, endocytosis of bacteria can be mediated through different uptake mechanisms that are either regulated by invasion proteins expressed by the bacterial pathogen or by the ability of the epithelial cells to engulf and potentially kill bacteria (Cossart and Helenius, 2014). Endocytosis of individual bacteria have been shown to use a receptor-mediated, zipper mechanism (clathrin- and actin-mediated internalization process), a trigger mechanism (a macropinocytosis-related process) or other unknown mechanisms (Cossart and Helenius, 2014). Uptake of bacterial aggregates in *Bartonella henselae* into an invasome using actin, talin-1 and β1-integrins have been observed in endothelial cells (Dehio et al., 1997; Eicher and Dehio, 2012). For GAS, uptake of planktonic bacteria through clathrin-mediated endocytosis (CME) occur by direct adherence or by using fibronectin as a bridge between the GAS surface protein (protein F1) and the host integrin (Ozeri et al., 2001; Kreikemeyer et al., 2004; Logsdon et al., 2011). Expression of SLO was found to modulate and effectively inhibit uptake of GAS through this mechanism (Logsdon et al., 2011). Mechanisms involved in uptake of biofilm bacteria have not been studied previously. Depending on the uptake mechanism used, endocytosed bacteria could end up in different intracellular compartments such as endosomes, lysosomes or other vacuoles. The acidic pH and toxic molecules (reactive oxygen or nitrogen species and acid proteases) in these compartments mediate bacterial elimination. However, some bacteria adapt or modify the vacuolar environment and stay there, whereas others manage to escape these vacuoles and persist within the cytosol for a longer duration of time (Hakansson et al., 2005; Kumar and Valdivia, 2009; Barnett et al., 2013). What uptake mechanisms and downstream events are responsible for the prolonged persistence of GAS biofilm bacteria is not known.

The aim of this study was to investigate the role of biofilm formation, virulence factors, as well as cellular uptake pathways in the association, internalization and persistence of GAS bacteria within epithelial cells. The rationale was to better understand the potential mechanisms associated with upper respiratory tract infections and recurrent pharyngitis to potentially find better therapeutic targets for treatment of infections caused by GAS.

## Materials and methods

### Reagents

Reagents for preparation of chemically defined medium (CDM) were purchased from VWR and Sigma. The medium was prepared as previously described (van de Rijn and Kessler, 1980). Todd-Hewitt broth, yeast extract, paraformaldehyde (PFA), nocodazole, Pitstop 2, Wortmannin, and Dynasore were purchased from Sigma. RPMI 1640 medium, Fetal bovine serum (FBS), Trypsin and other cell culture reagents were purchased from Hyclone, Thermo-Fisher. Gentamicin, AlexaFluor 647-conjugated donkey anti-goat antibody, AlexaFluor 568-conjugated phalloidin, AlexaFluor 488-conjugated transferrin or cholera toxin subunit B, and Fluorescein-conjugated dextran 70,000 MW were purchased from Invitrogen, Thermo-Fisher Scientific. Cytochalasin D was purchased from Calbiochem. Nystatin was purchased from Alfa Aesar. Anti-GAS antibody was purchased from Abcam.

### Planktonic growth of bacteria

The bacterial strains used in this study are listed in **Table 1** (Alamiri et al., 2020). To grow bacteria, a glass tube containing Todd-Hewitt broth with 0.5% yeast extract (THY) was inoculated with bacteria streaked on a blood agar plate. The inoculated bacteria were incubated at 37°C until reaching an OD_600 nm_ of 0.6 (~10^8^ CFU/ml). To prepare bacterial suspension for the internalization assays, planktonic bacteria were centrifuged, and the pellet was re-suspended in RPMI 1640 medium supplemented with 2% FBS and 100 mM sodium pyruvate (sRPMI-2%).

**Table 1:** Strains used in this study.

Strain name	Reference name	Phenotype (gene)	Source
M1T1	GAS-5448	M type 1 wild type strain with low capsule expression	(Chatellier et al., 2000)
M1	SF370	M type 1 wild type strain	(Abbot et al., 2007)
Δ*emm1*	SF370 △*emm1*	M1 protein (*emm1*) deletion mutant in SF370	
M5	Manfredo	M type 5 wild type strain	(Johnsson et al., 1998)
Δ*emm5*	Manfredo △*emm5*	M5 protein (*emm5*) deletion mutant in Manfredo	
M6	JRS4	M type 6 wild type strain from June Scott	(Biswas and Scott, 2003)
M11	GAS-53	Erythromycin-resistant clinical isolate expressing an *ermB* methylase	(Alamiri et al., 2019)
M12	GAS-6	Erythromycin-resistant clinical isolate expressing a *mefA* + *msrD* efflux pump.	
M18	87-282	M type 18 wild type strain with high capsule expression	(Wessels et al., 1991)
M22	GAS-8	Erythromycin-resistant clinical isolate expressing a *mefA* + *msrD* efflux pump. This strain lacks the *hasABC* genes encoding hyaluronic acid capsule.	(Flores et al., 2012; Alamiri et al., 2019)
M77	GAS-125	Erythromycin-resistant clinical isolate expressing an *ermTR* methylase.	(Alamiri et al., 2019)
M89	GAS-128	Erythromycin-resistant clinical isolate expressing an *ermB* methylase.	
**M3** s**trains used**			
M3	950771 (GAS-771)	M type 3 wild type strain, medium encapsulated, from child with necrotizing fasciitis and sepsis	(Ashbaugh et al., 1998)
Δ*emm3*	355	M type 3 *(emm3)* deletion mutant derived from 950771	
Δ*hasA*	188	Non-encapsulated isogen of 950771 with a Km-insertion in *hasA*	
Δ*hasA*-GFP	188-GFP	Δ*hasA* expressing GFP under the control of a GuaB promoter	(Hakansson et al., 2005)
Δ*slo*	500	Streptolysin O *(slo)* deletion mutant derived from 950771	(Bricker et al., 2002)
Δ*hasAΔslo*	781	Non-encapsulated (deletion) and SLO-deficient (Km insertion) double mutant derived from 950771	(Hakansson et al., 2005)
Δ*hasAΔslo-GFP*	781-GFP	Δ*hasA*Δslo expressing GFP under the control of GuaB promoter	(Hakansson et al., 2005)
Δ*nga*	C3	NADase (*nga*) mutant of 950771	(Bricker et al., 2002)
*ΔngaΔslo*	C42	950771 with deletion of NADase (*nga*) and SLO (*slo*)	
Δ*speB*	241	Cysteine protease (*speB*) deficient mutant derived from 950771, Km insertion in *speB*	(Ashbaugh et al., 1998)
Δ*sagA*	775	950771 with deletion of 60bp in Streptolysin S, SLS (*sagA*)	(Sierig et al., 2003)

### Biofilm formation

Biofilm formation was performed as described previously (Chao et al., 2019). A bacterial inoculum of ~10^5^-10^6^ CFU/ml of frozen bacterial stocks (in THY media with 13% glycerol, stored at -80°C) was first diluted in chemically defined medium (CDM, containing: 200 mg/L K_2_HPO_4_, 1,000 mg/L KH_2_PO_4_, 4,500 mg/L Na-acetate (NaH_2_C_3_O_2_), 5,000 mg/L NaHCO_3_, 3,195 NaH_2_PO_4_-H_2_O, 7,350 mg/L Na_2_HPO_4_, 700 mg/L MgSO_4_-7H_2_O, 5 mg/L FeSO_4_-7H_2_O, 1 mg/L Fe(NO_3_)_2_-9H_2_O, 5 mg/L MnSO_4_, 1 mg/L of CaCl_2_-6H_2_O, 500 mg/L L-cystein, 100 mg/L each of DL-Alanine, L-Arginine, L-Aspartic acid, L-Cystine, L-Glutamic acid, Glycine, L-Histidine, L-Isoleucine, L-leucine, L-lysine, L-methionine, L-Phenylalanine, L-Proline, Hydroxy-L-Proline, L-Serine, L-Tryptophan, L- Tyrosine, and L-Valine, 200 mg/L each or L-Glutamine and L-Threonine, 0.2 mg/L p-Aminobenzoic acid, 0.2 mg/L Biotin, 0.8 mg/L Folic acid, 1 mg/L Niacinamide, 2.5 mg/L of ß-NAD+, 2 mg/L Panthotenate calcium salt, 1 mg/L Pyridoxal, 1 mg/L Pyridoxamine-HCl, 2 mg/L Riboflavin, 1 mg/L Thiamine-HCl, 0.1 mg/L Vitamin B12, 20 mg/L each of Adenine, Guanine and Uracil, 1,000 mg/L Choline chloride and 10,000 mg/L carbon source as described in detail in (Chao et al., 2019). Microwell plates (24-well) covered with H292 cells prefixed in 4% paraformaldehyde (PFA) and washed twice in PBS were then seeded with bacteria diluted in CDM and incubated at 34°C (in the presence of 5% CO_2_) over a period of 72 h to form biofilms. Medium was replaced every 12 h. To prepare bacterial suspension for the live cell infection assay, medium was discarded, and biofilm bacteria were re-suspended in sRPMI-2% as described in detail below.

### Handling of planktonic and biofilm cultures and determination of viable organisms

To determine the colony forming units per ml (CFU/ml) in the bacterial cultures, viable plate counts were performed. Planktonic bacteria grown in broth were used directly for infection experiments and MIC assays as described below. The bacterial culture was diluted in a 10-fold serial dilution series and each dilution was plated on blood agar plates, and the plates were grown overnight at 37°C, the number of colonies was counted for each dilution where the counts were between 10 and 200 colonies and these numbers were used to determine bacterial concentration in CFUs/ml. For biofilm bacteria, each biofilm was detached from the cells by pipetting and transferred to a microfuge tube. These bacteria were used directly for infection experiments and for MIC assays, as described below. However, further disruption of bacterial aggregates formed in the biofilms were needed to properly determine the bacterial concentration. Therefore, mechanically disrupted biofilms were vortexed extensively and then exposed to a sonication bath for pulses of 2 seconds to break up potential aggregates, vortexed again, and the bacterial suspension was inspected in a microscope to ensure that no bacterial aggregation was observed before diluted and plated as described for planktonic bacteria above.

### Determining minimal inhibitory concentration

Minimal inhibitory concentration (MIC) was determined in 96-well microtiter plates using the microdilution method according to approved standards from the Clinical Laboratory and Standards Institute (CLSI, (Fuchs et al., 2002)) with some modifications. Rather than using the standard Mueller-Hinton broth, THY was used as it is a common media used for Streptococcal growth and used in the assays of the study. As indicated in prior studies, THY produced reliable MIC values for Streptococci (Marks et al., 2013; Alamiri et al., 2019). To test gentamicin and penicillin G susceptibility of planktonic and biofilm bacteria, a two-fold dilution series of each antibiotic of interest was prepared in duplicate wells for each of three cultures of planktonic or biofilm bacteria, respectively. Planktonic bacteria were used directly from the culture and diluted as indicated below. Biofilm bacteria were mechanically disrupted by pipetting off the biofilm from the cells and transferred to a microfuge tube. The tubes with bacterial suspensions were then vortexed and diluted for MIC testing. Each well was then seeded with a bacterial concentration of approximately 10^5^ CFU/mL in a total volume of 250 µl THY, and the plate was incubated for 18 h at 37°C in a Synergy 2 microplate reader (Biotek, Winooski, VT). The OD_600 nm_ was recorded every 10 minutes to monitor bacterial growth. The MIC was determined as the lowest concentration where no growth was detected.

### Epithelial cell culture

A liquid nitrogen frozen stock of the bronchial mucoepidermoid carcinoma cell line NCI-H292 from ATCC (ATCC®CRL-1848™) was inoculated into small flasks containing sRPMI-10%, i.e., RPMI 1640 medium supplemented with 10% FBS, 100 mM sodium pyruvate and a mixture of penicillin (100 units/ml) and streptomycin (100 μg/ml) and cells were incubated at 37°C (with 5% CO_2_) and grown to confluency. Media was replaced every 72 h. For cell propagation, attached cells were dissociated by 0.25% Trypsin treatment, detached cells were diluted in sRPMI-10%, and seeded at 1:5 – 1:10 dilution in fresh media into 24-well plates or μ-slide 8 well glass bottom IBIDI plates, respectively, and further incubated until confluency (3-4 days). For cell fixation, confluent cells were treated with 4% PFA, incubated at room temperature (RT) for 1 h, and washed 4 times with PBS prior to biofilm formation experiments.

### Live cell infection

Live H292 cells were infected with a multiplicity of infection (MOI) ranging from 0.025 to 1 CFUs/cell (i.e. ~2.5 x 10^4^ to 10^6^ CFUs added to 10^6^ cells, respectively) of actively growing planktonic bacteria or 72 h mature biofilms for 2 h and 30 min at 34°C (with 5% CO_2_). After incubation, cells were washed twice with PBS. To eliminate extracellular bacterial growth, cells were treated with 500 µg/ml gentamicin and 20 µg/ml penicillin in sRPMI-2%, prewarmed to 34°C and further incubated for an additional 1 h and 30 min. In parallel, supernatants and lysates of infected but non-antibiotic treated cells were used to determine bacterial growth in the media and cell-associated bacteria, respectively. Supernatant of both non-antibiotic treated and antibiotic treated cells were centrifuged at 9,000 rpm for 2 min, the pellet was re-suspended with PBS, 10-fold serially diluted, and dilutions were spread on blood agar plates. The supernatant from antibiotic-treated cells was included to ensure that no extracellular bacteria survived the treatment. Cells from non- or antibiotic-treated cells were dissociated using 1% trypsin, diluted in PBS and pelleted by centrifugation at 9,000 rpm for 2 min. To determine the CFU of intracellular bacteria, cell pellets were lysed using 0.1% Triton X-100 and the lysates were vortexed, diluted (10-fold), and spread on blood agar plates to determine CFUs, after incubation of the plates at 37°C for 48 h. Internalization efficiency, the percentage of total cell-associated bacteria that were internalized, was also calculated, and presented together with total cell numbers.

### Intracellular persistence

Cells infected with bacteria were treated with gentamicin and penicillin after 2 h and 30 min, as described above. Infected cells were then incubated with antibiotics for a total of 4 h, 7 h, 20 h, or 44 h at 34°C. Supernatants of cells treated or non-treated with antibiotics were collected, centrifuged, and stored at -80°C for cytotoxicity analysis. The bacterial pellet (dissolved in PBS) as well as cell lysates from each time point were spread on blood agar plates (as described above) to assess the CFU of bacterial growth in media or internalized bacteria, respectively, at each time point after incubation of the plates at 37°C for 48 h.

### Uptake inhibition assay

To determine the cellular uptake pathways involved in the internalization of GAS bacteria, live H292 cells were treated for 1 h with inhibitors targeting uptake pathways involved in bacterial uptake such as cytochalasin D (inhibits actin, 50 μg/ml), nocodazole (inhibits microtubulin, 10 μg/ml), pitstop 2 (inhibits clathrin mediated uptake, 11.8 μg/ml), Dynasore (inhibits dynamin, 25.7 μg/ml), nystatin (inhibits lipid-raft mediated uptake, 7.5 μg/ml), and wortmannin (inhibits micropinocytosis, 100 µg/ml). The media was removed, and the inhibitor-treated cells were inoculated with planktonic or biofilm GAS-771 bacteria at an MOI of 1 for 4 h in media containing fresh inhibitors and the CFU’s of internalized and cell-associated bacteria in the presence or absence of inhibitors were determined (as mentioned above). To determine the inhibition activity of the inhibitors, inhibitor-treated cells were exposed to 25 μg/ml transferrin or 4 μg/ml cholera toxin subunit B conjugated to AlexaFluor-488, or 50 μg/ml of Dextran with a molecular weight of 70,000 conjugated to fluorescein for 20 min, 1 h, or 2 h, respectively, and further visualized using a Zeiss Axiovert.A1 microscope (Zeiss).

### Cytotoxicity assay

Cells were stimulated with GAS suspended in sRPMI-2% or with RPMI 1640 medium alone (spontaneous release) and all cells were treated with antibiotics (500 µg/ml gentamicin and 20 µg/ml penicillin in sRPMI-2% prewarmed to 34°C) after 2.5 hours of incubation. Supernatants from GAS or media-alone treated cells, or cells exposed to lysis solution (to measure maximum release) were collected at indicated timepoints and stored at -80°C. The amount of released Lactate dehydrogenase (LDH) was measured using the CyQUANT™ LDH cytotoxicity assay kit (Thermo Fischer Scientific). The color intensity was analyzed using the Synergy 2 microplate reader (Biotek, Winooski, VT) at 490 nm and the percentage of cytotoxicity was calculated following the formulas in the provided protocol.

### Sample preparation for imaging

Live H292 cells grown on a μ-slide 8 well glass bottom (Ibidi, Munich, Germany) and were infected with biofilm and planktonic bacteria of GFP-expressing GAS strains (Δ*hasA* or Δ*hasA*Δ*slo*) using an MOI of 10. The media was removed, cells were washed in PBS (1X) and fixed by 4% PFA as described above. Extracellular bacteria were stained using a primary goat anti-GAS antibody (3.2 µg/ml) diluted 1:500 in 0.05% Bovine serum albumin (BSA) for 30 min. Excess staining was removed by washing in PBS (1X) three times. Secondary AlexaFluor 647 conjugated donkey anti-goat antibody (Invitrogen™, Thermo Fischer Scientific) diluted 1:500 in PBS (1X) was then added for 30 min in the dark. Excess antibody was removed by washing in PBS (1X) three times. Subsequently, cells were permeabilized using Triton X (0.01%) for 15 min at 37°C, washed, and stained using AlexaFluor 568 phalloidin (Invitrogen) using a 1:500 dilution in PBS (1X). DNA was stained by using Hoechst (Thermo Fischer Scientific) diluted to a working concentration of 1 µM in PBS for 30 min and washed for in PBS prior to imaging.

### Fluorescence microscopy

Images of internalized and extracellular bacteria were acquired using a widefield epifluorescent Nikon Ti2-E inverted microscope with Nikon SR Plan Apo IR 60xAC WI, NA = 1.27. Fluorescence was excited with the SPECTRA X light engine^®^ (Lumencore Inc, Beaverton, OR, USA) and collected with DAPI (Exc. 379-405 nm, Em. 414-480 nm), FITC (Exc. 457-487 nm, Em. 503-538 nm), TRITC (Exc. 543-566 nm, Em. 582-636 nm) and Cy5 (Exc. 590-645 nm, Em. 659-73 6nm) filter cubes, all from Semrock. Images were acquired using Nikon DS-Qi2 CMOS controlled with NIS-Elements (v. 5.21.02). Multiple stage positions were collected using a Nikon motorized stage. For each data set, 3x3 20X magnification images were first collected, including z-stacks, allowing them to cover a wide area of the sample. From these images, multiple areas of interest were imaged at 60X and deconvolved using NIS-Elements (v 5.21.02) to remove out-of-focus light. After analysis of the deconvolved images, representative images were selected. Image gamma, brightness, and contrast were adjusted using NIS-Elements Viewer.

### Statistical analysis

GraphPad Prism 9 software (GraphPad Software LLC) was used to graph data and perform statistical analyses. Internalization, persistence, inhibitor treatment LDH cytotoxicity were analyzed using Student’s t-test or one-way ANOVA with Dunnett’s multiple comparison tests. *P*-values of <0.05 were considered statistically significant. Data are presented as mean values, with error bars representing the standard deviation, i.e., the error of mean values of multiple biological data points as described in detail in the figure legend for each experiment. Simple linear regression was used to analyze the persistence for each infection (Log_10_ CFU/ml over time) as well as the significant correlation between bacterial intracellular survival curves between bacterial populations and strains.

## Results

### Association, internalization and persistence of GAS biofilm and planktonic bacteria in epithelial cells

**Figure 1:**
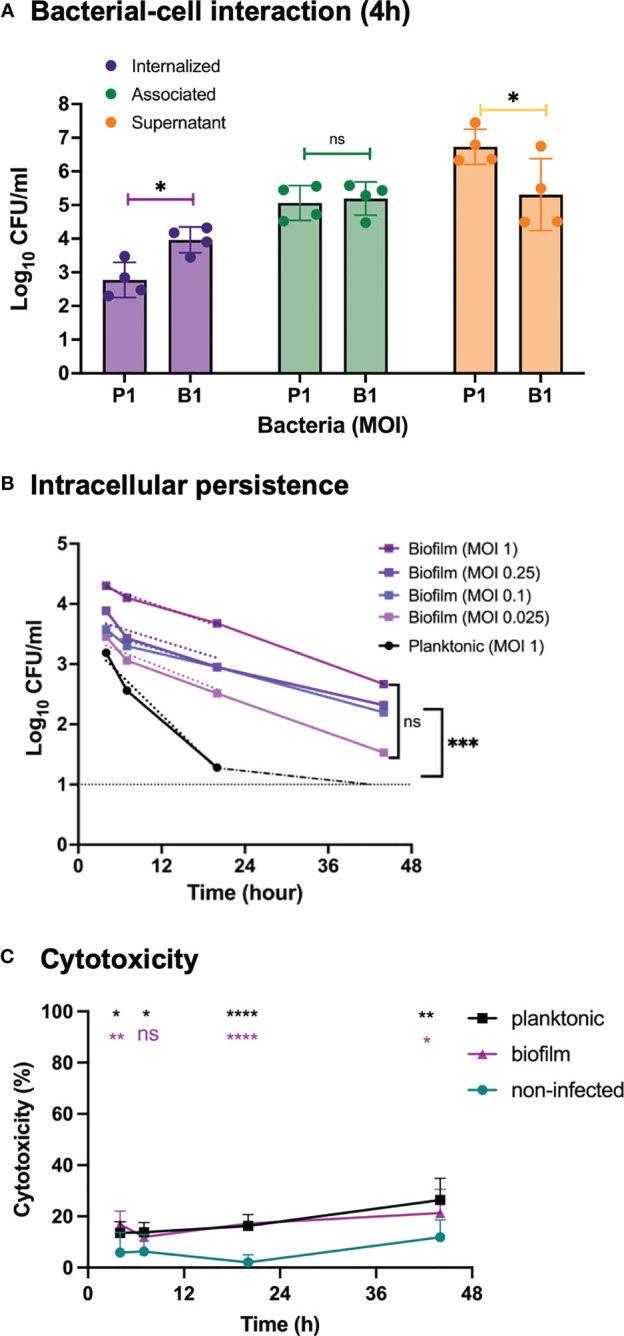
Bacterial growth, cell association, internalization and persistence and inflammatory response in respiratory epithelial cells exposed to *Streptococcus pyogenes*. Live respiratory epithelial (H292) cells were infected with *S. pyogenes* strain GAS-771 grown planktonically or as biofilms. **(A)** Cells were inoculated with bacteria at 34°C for 4 h without antibiotic treatment, or 2.5 h followed by antibiotic treatment for 1.5 h to eliminate extracellular bacteria. Bacterial growth in the culture supernatant (Supernatant, orange bars), total association to the cells (Association, green bars), or internalization levels (Internalization, purple bars) were assessed by determining the Log_10_ CFUs, 4 h post infection. P1 and B1 indicate inoculation of bacteria with planktonic (P) and biofilm **(B)** bacteria at a multiplicity of infection (MOI) of 1. The results represent mean data from four separate experiments ± standard deviation (SD) (n = 4). Differences in supernatant growth (orange), association (green), or internalization (purple), between planktonic and biofilm bacteria was compared using one-way ANOVA using Dunnett’s multiple comparison tests. **(B)** Persistence of intracellular planktonic (MOI 1; black line) or biofilm bacteria (MOIs of 0.025-1; blue to purple lines) was measured by assessing the Log_10_ CFU in lysates of antibiotic treated cells at 4, 7, 20, or 44 h post infection and displayed as mean data ± SD (n = 4 experiments). The elimination rates of intracellular bacteria were determined by linear regression analysis for Log_10_ CFU of intracellular bacteria over time (dotted lines from 0-20 h). As indicated, no significant difference was observed between the elimination rates of the various MOIs of biofilms. The significant difference in elimination rates between planktonic MOI 1 and biofilm MOI of 0.025 is shown to the right. **(C)** To assess the cell viability, the cytotoxicity percentage of antibiotic treated and uninfected cells (green line) or cells infected with planktonic (black line) or biofilm bacteria (purple line) at an MOI of 1, was determined by measuring the release of lactate dehydrogenase (LDH) into the culture supernatant during antibiotic treatment. Data is displayed as mean ± SD for n=9 experiments. Differences between untreated cells and planktonic bacteria or biofilm bacteria were compared using one-way ANOVA with Dunnett’s multiple comparison tests. Significance was displayed with black (planktonic) or purple (biofilms) stars. For all statistical analyses *P < 0.05, **P < 0.01, ***P < 0.001, ****P < 0.0001, and ns, non-significant difference.

Uptake of broth-grown, planktonic GAS into eukaryotic cells have been previously described (LaPenta et al., 1994; Greco et al., 1995; Cue et al., 2000; Ozeri et al., 2001; Marouni et al., 2003; Staali et al., 2003; Hakansson et al., 2005; Logsdon et al., 2011). Using an *in vitro* cell infection model, we have also demonstrated the ability of biofilm bacteria to both internalize in higher numbers and persist longer than planktonic bacteria inside keratinocytes (Marks et al., 2014). To investigate bacterial association to and uptake into epithelial cells, as well as their intracellular persistence we initially used *S. pyogenes* strain GAS-771 (M3) that has previously been used to study biofilm formation and host-cell interactions (Hakansson et al., 2005; Logsdon et al., 2011; Alamiri et al., 2020). Live respiratory epithelial cells (H292 cells) were inoculated with actively growing planktonic (late logarithmic phase) or biofilm bacteria (grown for 72 h at a nasopharyngeal temperature of 34°C) for various times. A multiplicity of infection (MOI) of 1 colony forming unit (CFU) per cell was used to obtain a measurable uptake of planktonic and biofilm bacteria (**Figure 1A**, purple bars) without causing major cell cytotoxicity (**Figure 1C**).

Lysates and cell supernatant of non-antibiotic treated cells were used to determine the total level of association of GAS bacteria to cells and the bacterial growth in the cell medium, respectively (**Figure 1A**, green and orange bars, respectively). Live intracellular bacteria were determined from infected cells treated with penicillin and gentamicin to eliminate extracellular bacteria prior to plating the cell lysates. (**Figure 1A**, purple bars). GAS-771 biofilm bacteria showed significantly lower CFU counts than planktonic cells in the cell supernatant, suggesting slower growth (**Figure 1A**, orange bars), but still displayed similar total bacterial association levels to epithelial cells. Despite similar total bacterial association (**Figure 1A**, green bars), approximately ten times (11.1 times) more biofilm than planktonic bacteria were found viable inside the epithelial cells after 4 h (**Figure 1A**, purple bar). This corresponded to an internalization efficiency (percent of total cell-associated bacteria found viable on the inside of the cells) of 0.56% for planktonic bacteria and 6.15% for biofilm bacteria. To ensure that this increase in intracellular biofilm bacteria was not a result of an increased tolerance to antibiotics of biofilm bacteria, two assays were performed. First, the supernatant from antibiotic treated cells were plated to ensure that no surviving organisms were detected. Second, the MIC for penicillin and gentamicin of biofilm and planktonic GAS-771 bacteria were determined. The MIC values for penicillin G and gentamicin were 0.016 µg/ml and 1 µg/ml for planktonic bacteria respectively. Interestingly, the corresponding MIC values for biofilm bacteria were 2- to 4-fold lower (0.008 µg/ml and 0.25 µg/ml, respectively), suggesting that biofilm bacteria were more sensitive to antibiotics in this assay.

Intracellular persistence was determined by allowing internalization for 2.5 h, killing extracellular bacteria, and measuring the number of live intracellular bacteria over time, at 4 h, 7 h, 20 h, and 44 h post infection (**Figure 1B**). At an MOI of 1, the internalized biofilm bacteria persisted throughout the experiment at significantly higher levels and were killed at a significantly slower rate inside the cells than planktonic bacteria that were eliminated and undetectable prior to 20 h (**Figure 1B**; *P* < 0.001). To control for the higher intracellular viability of biofilm bacteria at 4 hours, titration of the inoculum of biofilm bacteria was performed. Epithelial cells were inoculated with an MOI of 0.25, 0.1 and 0.025 of biofilm bacteria and intracellular viability of bacteria and persistence were measured. An MOI of 0.25 of biofilm bacteria still resulted in significantly higher intracellular CFU counts than for planktonic bacteria, whereas both an MOI of 0.1 and 0.025 resulted in intracellular CFU counts that were not significantly different from bacteria inoculated with planktonic bacteria at an MOI of 1 (**Figure 1B**). Yet, clearance of bacteria over time was significantly slower for biofilm bacteria also at these MOIs compared with planktonic organisms (**Figure 1B**), suggesting that intracellular starting concentration was not a deciding factor for bacterial intracellular clearance but that the biofilm phenotype was important for protection against intracellular elimination. This difference was not correlated with differences in cell cytotoxicity that was induced at similar levels by both biofilm and planktonic GAS over time (**Figure 1C**). Combined, these results confirm earlier findings showing higher internalization and persistence rates of GAS biofilm bacteria than planktonic bacteria also within respiratory epithelial cells.

### Association, internalization and persistence of other M types in epithelial cells

**Figure 2:**
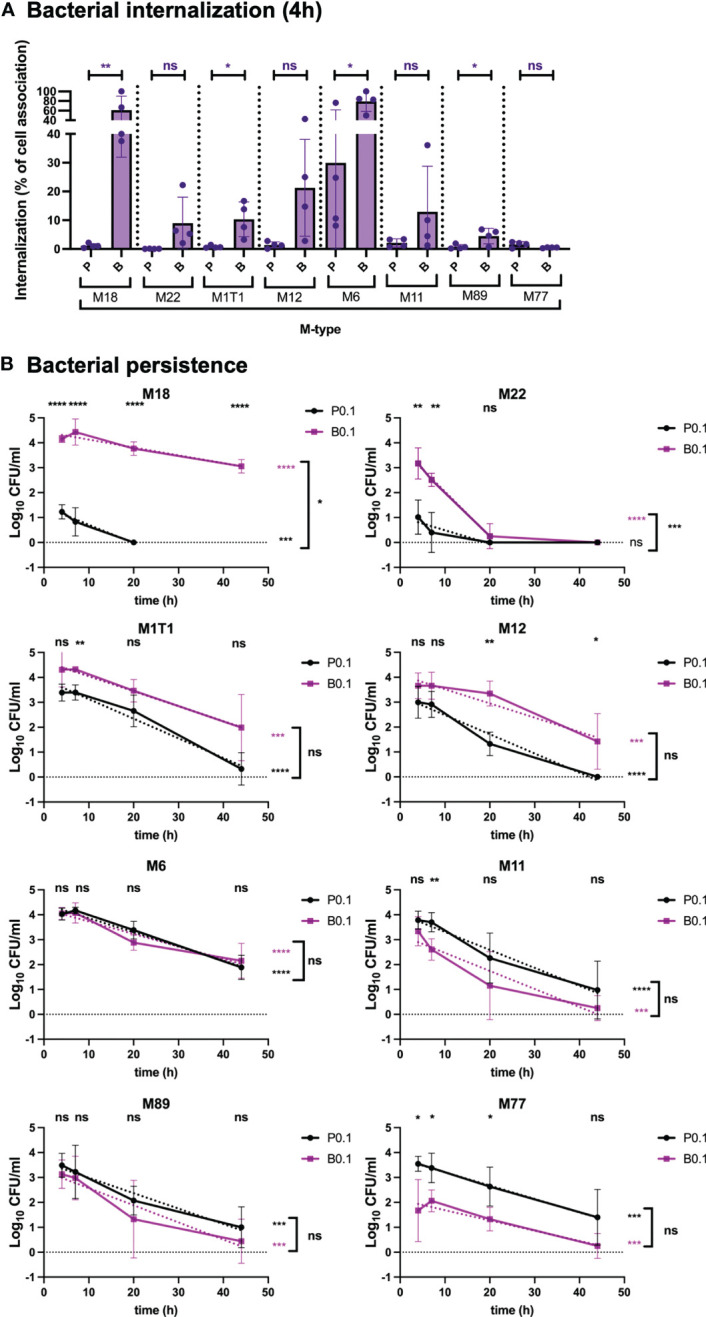
Bacterial internalization and persistence of different GAS serotypes in respiratory epithelial cells. Live respiratory epithelial (H292) cells were infected with *S. pyogenes* M1T1, M6, M11, M12, M18, M22, M77 or M89 grown planktonically or as biofilms. **(A)** Cells were inoculated with bacteria at 34°C for 2.5 h followed by antibiotic treatment for 1.5 h to eliminate extracellular bacteria or for 4 h without antibiotics to assess total cell association. Bacterial internalization and cell-association levels were assessed by determining the Log_10_ CFUs, 4 h post infection (data shown in **Figure S2**). Internalization was then presented in the graph as the percentage of cell-associated bacteria that were internalized for each experiment (purple). P and B indicate inoculation of bacteria with planktonic (P) or biofilm **(B)** bacteria at a multiplicity of infection (MOI) of 0.1. The results represent mean data from four separate experiments ± SD (n = 4) with individual data points presented in the graph. Differences in internalization between planktonic and biofilm bacteria for each M type was compared using one-way ANOVA using Dunnett’s multiple comparison test and is displayed on top of each bar. **(B)** Persistence of intracellular planktonic (black line) or biofilm (purple line) bacteria was measured by assessing the Log_10_ CFU in lysates of antibiotic treated cells at 4, 7, 20, or 44 h post infection and displayed as mean data ± SD (n = 4 experiment). P0.1 and B0.1 indicate inoculation of bacteria with planktonic (P) or biofilm **(B)** bacteria at a multiplicity of infection (MOI) of 0.1. The difference between internalization levels of planktonic and biofilm bacteria for each timepoint was evaluated using Student’s t-test (shown on top of each time point in black). The elimination rates of intracellular bacteria were determined by assessing the linear correlation between Log_10_ CFU of intracellular bacteria over time (dotted lines). Linear coefficients significantly different from zero are presented in purple or black stars next to each line. Significant differences in elimination rates between intracellular planktonic and biofilm bacteria is shown to the right of bracket in black. For all statistical analyses *P < 0.05, **P < 0.01, ***P < 0.001, ****P < 0.0001 and ns, non-significant difference.

To determine whether the uptake and persistence phenotypes observed for GAS-771 (M3) were generally observed for GAS strains, respiratory epithelial cells were infected with biofilm and planktonic bacteria from clinical isolates of different M types (M1T1, M6, M11, M12, M18, M22, M77 and M89). Except for the M18 isolate, total cell association was similar or significantly higher for planktonic bacteria than for biofilm bacteria, especially in the M11 and M89 strains (**Figure S2**). Like for GAS-771 (**Figure 1A**), higher absolute internalization levels were detected for biofilm bacteria than for planktonic bacteria for the M18, M22, M1T1 and M12 isolates with internalization being significant higher for M18 and M22 (**Figure S2**, purple bars). In contrast, total internalization levels of biofilm bacteria were the same as for planktonic bacteria after inoculation with strains M6, M11, M89, and significantly lower for the M77 strain (**Figure S2**, purple bars). However, when considering the internalization efficiency (the percentage of cell-associated bacteria found viable in the intracellular environment), two observations were made. First, internalization levels were higher for biofilm bacteria than for planktonic bacteria for all M-types except M77, with the internalization efficiency being significant higher for M18, M1T1, M6 and M89 and higher for M22 and M12 (*P* = 0.09 and 0.06, respectively) (**Figure 2A**). Strain M77 showed low internalization with no significant differences in uptake as a percentage of cell-association. Second, planktonic bacteria of all serotypes except M6 (mean internalization efficiency 29.9%) showed low internalization efficiencies (0.05-2.11%), whereas biofilm bacteria were generally internalized more efficiently (up to 79% of cell associated bacteria), with M18 and M6 showing particularly high internalization efficiency (61 and 79%, respectively) (**Figure 2A**).

When investigating persistence of bacteria in the intracellular environment, strain M18 showed a similar phenotype as the M3 strain GAS-771 (**Figure 1B**), with biofilm bacteria persisting at significantly higher numbers for over 44 h, whereas planktonic bacteria were eliminated within 20 h at a significantly higher rate (**Figure 2B**). For all remaining strains, except for the M22 strain, the rate of elimination of intracellular bacteria over time was not significantly different between biofilm and planktonic bacteria and most strains had viable organisms remaining after 44 h (**Figure 2B**). Strain M22, showed a pattern where both biofilm bacteria and planktonic bacteria persisted poorly, and were eliminated from the intracellular environment within the first 20 h (**Figure 2B**). As observed for the M3 (GAS-771) strain (**Figure 1C**), cellular viability was not affected by the presence of these bacterial isolates during the first 4 h of infection (**Figure S1A**).

Combined, these results show that GAS uptake into respiratory epithelial cells was a general characteristic for all M types tested. However, cell association, internalization and persistence levels were not identical between strains. Interestingly, although variability was observed between the strains, internalization of biofilm bacteria was high and varied less, in terms of total numbers, than the uptake of planktonic bacteria, suggesting that biofilm bacteria have a more homogenous internalization phenotype than bacteria grown in broth. Similarly, planktonic organisms showed low internalization efficiency in terms of percent of total cell-association, whereas the internalization of biofilm bacteria was more efficient.

### Role of the M protein in cell association, uptake and persistence of planktonic and biofilm bacteria in epithelial cells

**Figure 3:**
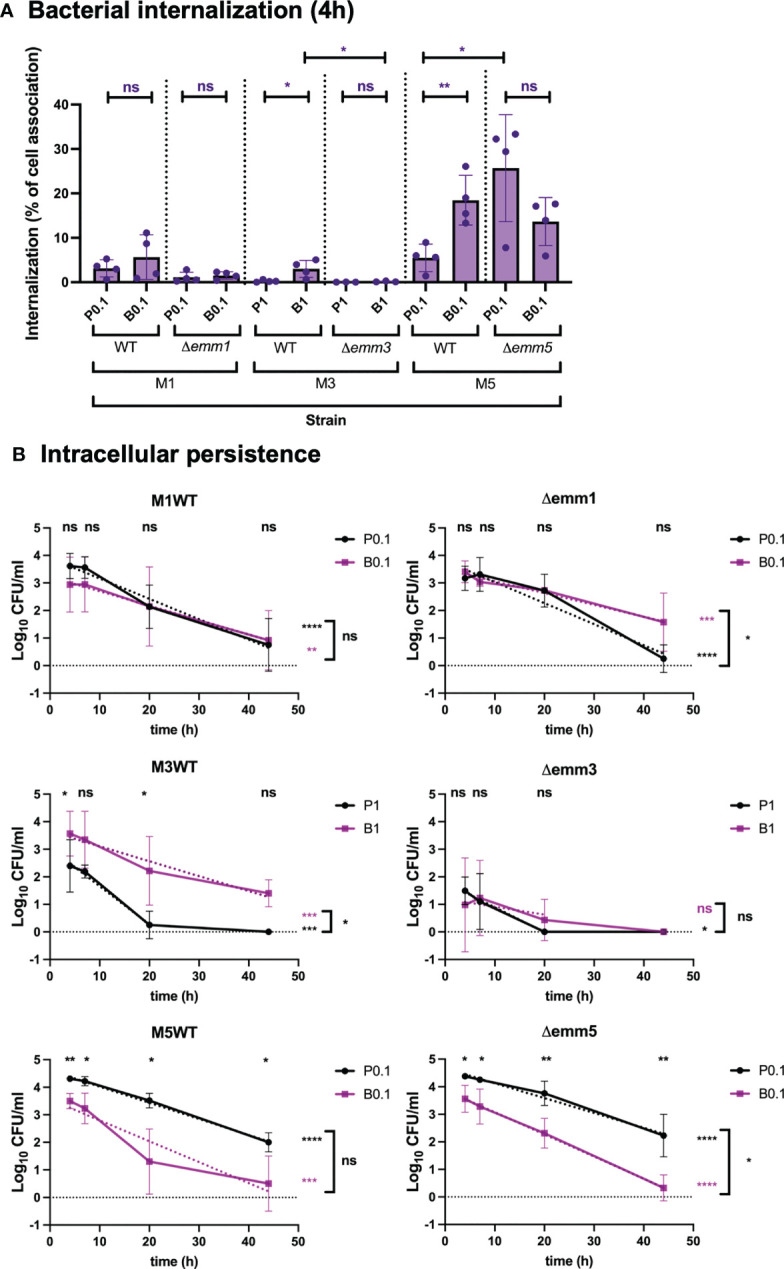
Role of the M protein in GAS bacterial internalization and persistence during respiratory epithelial cell infection. GAS M1 (SF370), M3 (GAS-771) and M5 (Manfredo) strains expressing (WT) or lacking the M protein (Δ*emm*) and grown planktonically or as biofilms were used to infect live respiratory epithelial (H292) cells. **(A)** Cells were inoculated with bacteria at 34°C 2.5 h followed by antibiotic treatment for 1.5 h to eliminate extracellular bacteria or for 4 h without antibiotics to assess total cell association. Bacterial internalization and cell-association levels were assessed by determining the Log_10_ CFUs, 4 h post infection (data shown in **Figure S3**). Internalization was then presented in the graph as the percentage of cell-associated bacteria that were internalized for each experiment (purple). P1 (or P0.1) and B1 (or B0.1) represent planktonic (P) or biofilm (P) bacteria used at a multiplicity of infection (MOI) of 1 (or 0.1). The results represent mean data from four (or three in Δ*emm3*) separate experiments ± SD. Differences internalization between planktonic and biofilm bacteria in each strain, or between WT and Δ*emm*, was compared using one-way ANOVA using Dunnett’s multiple comparison tests and is displayed by stars in the graph. **(B)** Persistence of intracellular planktonic (black line) or biofilm (purple line) bacteria was measured by assessing the Log_10_ CFU in lysates of antibiotic treated cells at 4, 7, 20, or 44 h post infection and displayed as mean data ± SD (n = 4 experiments for all strains except n = 3 in Δ*emm3*). Differences between internalization levels of planktonic and biofilm bacteria for each timepoint was evaluated using Student’s t-test (stars shown on top of each time point in black). The elimination rates of intracellular bacteria were determined by determining the linear correlation between Log_10_ CFU of intracellular bacteria over time (purple and black dotted lines for planktonic and biofilm bacteria, respectively). Linear coefficients significantly different from zero are presented in purple or black stars next to each line. Significant differences in elimination rates between intracellular planktonic and biofilm bacteria is shown to the right of bracket in black. For all statistical analyses *P < 0.05, **P < 0.01, ***P < 0.001, ****P < 0.0001 and ns, non-significant difference.

M protein is known for its essential role as an adherence factor in GAS (Brouwer et al., 2016). The M1, M3 and M5 proteins have been shown previously to promote adherence of GAS to human cells and the M1 and M3 (but not M5) proteins have been shown to be involved in the uptake of planktonic GAS bacteria into endothelial and epithelial cells (Courtney et al., 1997; Eyal et al., 2003; Ochel et al., 2014). To determine the role of the M protein during uptake of biofilm bacteria, GAS M1 (SF370), M3 (GAS-771) or M5 (Manfredo) expressing or lacking the M protein gene (*emm*) was used to infect respiratory epithelial cells (**Figure 3** and **S3**) at concentrations that did not induce cytotoxicity above untreated control cells (**Figure S1B**). In contrast to previous studies, no direct role of the M1, M3 or M5 proteins was observed in cell association of GAS to respiratory epithelial cells over a 4-hour incubation period (**Figure S3**). Internalization of planktonic M1 (SF370) bacteria into epithelial cells did not differ significantly from biofilm bacteria and the absence of biofilm did not affect internalization, either in terms of total numbers (**Figure S3**, purple bars) or as a percentage of total cell-association (**Figure 3B**) and both bacterial phenotypes from associated and persisted intracellularly equally well (> 44 h) (**Figure S3**, **Figure 3B**). However, the absence of the M1 protein resulted in a slower initial elimination rate of biofilm bacteria over 44 h (**Figure 3B**).

Like previous results (Eyal et al., 2003), absence of the M3 protein (Δ*emm3*) resulted in a significant reduction of internalization of biofilm bacteria after 4 h compared to its WT M3 strain (GAS-771) despite similar cell association, both in terms of total numbers (**Figure S3**, purple bars) and as a percentage of total cell-association (**Figure 3B**). A reduction of total numbers was also seen for M3-negative planktonic bacteria, but to a less, and non-significant extent (**Figure S3**, purple bars). Lack of the M3 protein also resulted in a shorter intracellular persistence of biofilm bacteria (<44 h) as compared to its WT strain (>44 h), whereas the elimination rate was similar for planktonic bacteria, irrespective of M3-expression (**Figure 3B**).

Interestingly, like some M types in **Figure 2** and **S2**, planktonic bacteria of the M5WT (Manfredo) isolate both associated and internalized in significantly higher total numbers than biofilm bacteria (**Figure S3**) and persisted at significantly higher numbers over 44 h (**Figure 3B**). However, due to the higher cell-association of planktonic bacteria, the internalization efficiency of M5WT biofilm bacteria, as a percentage of cell-associated bacteria was, in fact, significantly higher than for planktonic bacteria (**Figure 3A**). The lack of the M5 protein did not dramatically affect the cell-association or total internalization (**Figure S3**) but showed higher percentage internalization than the M5WT (**Figure 3A**). Similarly, the lack of the M5 protein did not affect persistence of either planktonic or biofilm bacteria inside the cells over time, other than that, for this strain, biofilm bacteria were eliminated at a slightly higher rate than its planktonic counterpart (**Figure 3B**).

These results indicate that only M3 protein expression was required for optimal internalization and persistence of intracellular bacteria in our epithelial cell model. In contrast, neither M1 nor M5 protein were needed to associate with epithelial cells or to escape cell-mediated killing of intracellular planktonic and biofilm bacteria, respectively.

### Role of hyaluronic acid capsule and SLO during association, uptake and persistence in epithelial cells

The role of the hyaluronic acid capsule during GAS endocytosis is not clear. Some studies have indicated an essential role of capsule for colonization and invasion in *in vitro* and *in vivo* infection models (Wessels et al., 1991; Ashbaugh et al., 1998). Still, high levels of capsule expression are thought to inhibit internalization into epithelial cells as GAS mutants lacking capsule internalize in higher numbers than their corresponding WT strains (Schrager et al., 1996; Jadoun et al., 2002; Hakansson et al., 2005). In support of this, our results in **Figures 1**, **2** show that planktonic bacteria of strains expressing low (M6 and M1T1), medium (M3) or high (M18) levels of capsule (**Table 2**) showed inverse (high, medium or low, respectively) levels of total bacterial uptake (**Figures 1**, **S2** **)**. However, this pattern was not observed for biofilm bacteria. Similar to capsule, SLO has previously been shown by us to play an inhibitory role during GAS internalization of planktonic bacteria into keratinocytes (Schrager et al., 1996; Jadoun et al., 2002; Hakansson et al., 2005).

**Table 2:** Quantification of hyaluronic acid capsule levels in GAS isolates.

Strain name	Capsule amount (fg/CFU)*
M1T1	0.108
M3 (GAS-771)	8.44
M6	0.128
M18	81.096

* Data from (Alamiri et al., 2020).

**Figure 4:**
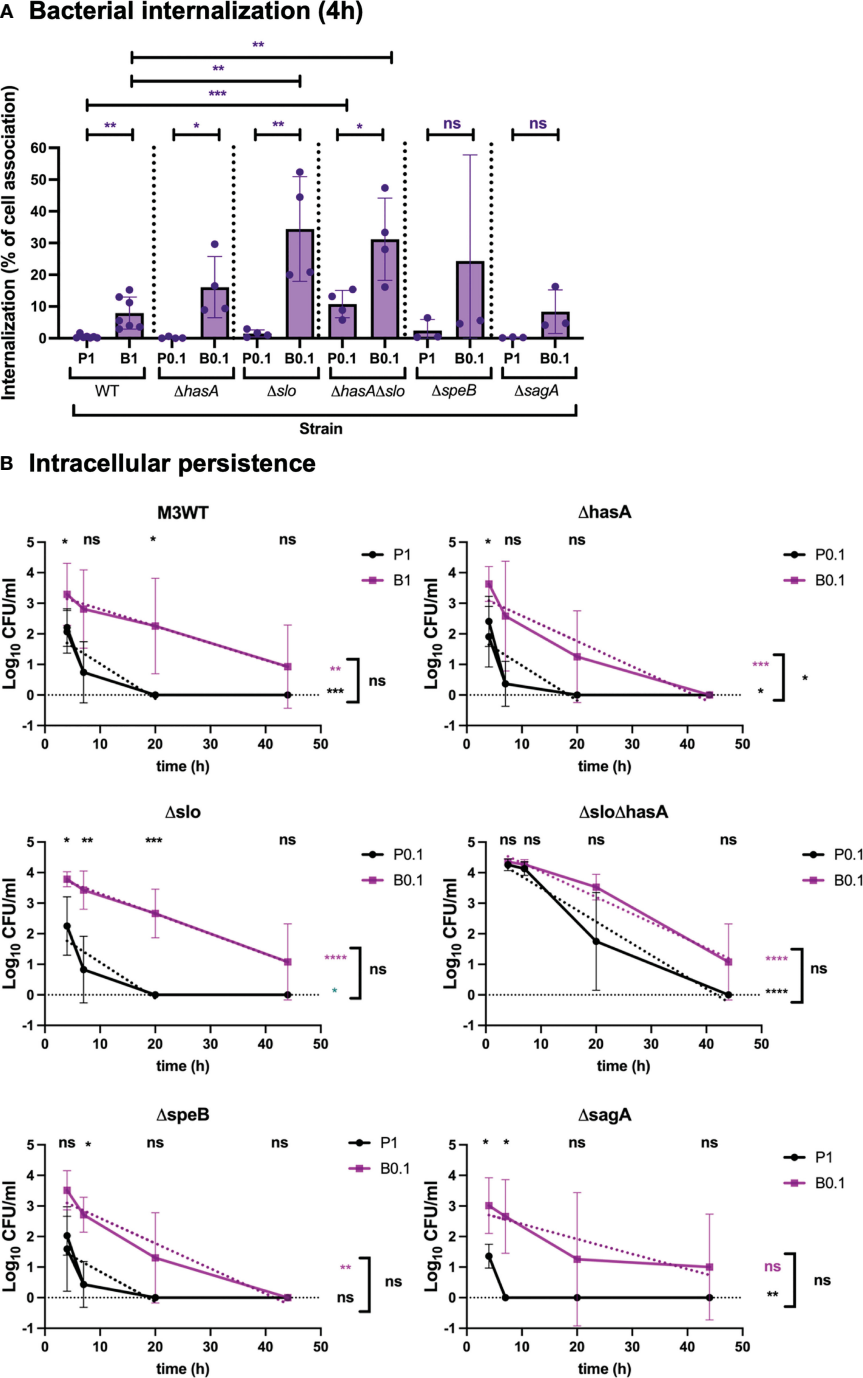
Role of virulence factors in GAS internalization and persistence during respiratory epithelial cell infection. To determine the role of virulence factors during GAS infection, M3 (GAS-771) strain expressing wild-type (WT) or lacking capsule (Δ*hasA*), SLO (Δ*slo*), or both (Δ*hasA*Δ*slo*), or SpeB (Δ*speB*), or SLS (Δ*sagA*) grown planktonically or as biofilms, were used to infect live respiratory epithelial (H292) cells. **(A)** Cells were inoculated with bacteria at 34°C for 2.5 h followed by antibiotic treatment for 1.5 h to eliminate extracellular bacteria or for 4 h without antibiotics to assess total cell association. Bacterial internalization and cell-association levels were assessed by determining the Log_10_ CFUs, 4 h post infection (data shown in **Figure S4**). Internalization was then presented in the graph as the percentage of cell-associated bacteria that were internalized for each experiment (purple). P1 (or P0.1) and B1 (or B0.1) represent planktonic (P) and biofilm **(B)** bacteria a multiplicity of infection (MOI) of 1 (or 0.1). The results represent mean data from three (in Δ*speB*, or Δ*sagA*), four (in Δ*hasA*, Δ*slo*, or Δ*hasA*Δ*slo*), or seven (M3WT), separate experiments ± SD (n = 3 experiments in Δ*speB*, or Δ*sagA*, or n = 4 experiments in Δ*hasA*, Δ*slo*, or Δ*hasA*Δ*slo*, or n = 7 experiments in M3WT). Differences internalization between planktonic and biofilm bacteria in WT and mutant strains was compared using one-way ANOVA using Dunnett’s multiple comparison tests and are shown on top of each bar, and the difference between planktonic and biofilm bacteria is shown on top of the black line. **(B)** Persistence of intracellular planktonic (black line) or biofilm (purple line) bacteria was measured by assessing the Log_10_ CFU in lysates of antibiotic treated cells 4, 7, 20, or 44 h post infection and displayed as mean data ± SD (n = 3 experiments in Δ*speB*, or Δ*sagA*, or n = 4 experiments in Δ*hasA*, Δ*slo*, or Δ*hasA*Δ*slo*, or n = 7 experiments in M3WT). Significant differences between internalization levels of planktonic and biofilm bacteria for each timepoint was evaluated using Student’s t-test (shown on top of each time point in black). The elimination rates of intracellular bacteria were determined by assessing the linear correlation between Log_10_ CFU of intracellular bacteria over time (dotted lines). Linear coefficients significantly different from zero are presented in purple or black stars next to each line. The significant difference in elimination rates between intracellular planktonic and biofilm bacteria is shown to the right of bracket in black. For all statistical analyses *P < 0.05, **P < 0.01, ***P < 0.001, ****P < 0.0001 and ns, non-significant difference.

To address the direct role of capsule and SLO during internalization and persistence of GAS, live respiratory epithelial cells were infected with M3WT (GAS-771) planktonic and biofilm bacteria lacking expression of the hyaluronic acid capsule (Δ*hasA*), SLO (Δ*slo*), or both (Δ*hasA*Δ*slo*) (**Figure 4** and **Figure S4**). To better be able to compare the internalization and persistence between WT and the strains lacking capsule, SLO or both, cells were inoculated with different MOIs in an attempt to reach similar levels of internalization after 4 hours (**Figure 4** and **Figure S4**). Infection performed at these MOIs did not induce cytotoxicity above untreated control cells (**Figure S1C**). For planktonic organisms, total internalization levels of the Δ*hasA* and Δ*slo* mutants reached similar levels as the WT strain inoculated at an MOI of 1 (**Figure S4**). When assessing internalization efficiency (percent internalization of total cell-association), similar results were observed with WT, Δ*hasA*, and Δ*slo* being taken up at 0.5, 0.2 and 1.5% of the total cell-association levels, respectively (**Figure 4A**). Even at the lower MOI of 0.1, the planktonic bacteria lacking both factors internalized at significantly higher levels than the WT strain (**Figure S4**), which was mirrored by the significantly increased internalization efficiency of 11% (**Figure 4A**).

For biofilm bacteria, the WT strain, the non-encapsulated Δ*hasA* strain, and the SLO-negative Δ*slo* strain showed both a significantly higher internalization efficiency and higher total internalization numbers than their respective planktonic bacteria (**Figure 4A** and **Figure S4**). Although no significantly increased number of internalized bacteria was detected (**Figure S4**), the internalization efficiencies were increased in both the Δ*hasA*, and Δ*slo* strains (**Figure 4A**). Finally, absence of both capsule and SLO resulted in higher internalization efficiency for biofilm bacteria than for planktonic bacteria (**Figure 4A**). This was not, however, observed when measuring total internalization numbers alone, probably due to the high internalization numbers of planktonic bacteria for this strain (**Figure S4**). Also, like the planktonic organisms, Δ*hasA*Δ*slo* bacteria were internalized at significantly higher efficiency than WT biofilm bacteria (**Figure 4A**).

Absence of capsule or SLO did not affect the elimination rate of intracellular planktonic bacteria that were eliminated within 20 h. The elimination of biofilm bacteria was slower for all strains, although somewhat more rapid for the capsule-negative biofilm bacteria (eliminated ≤ 44 h) than for the corresponding WT strain (survived > 44 h) (**Figure 4B**). On the other hand, differences in elimination rate were less clear for the mutant lacking expression of both capsule and SLO (Δ*hasA*Δ*slo*) that persisted for up to 44 hours both planktonic and biofilm bacteria (**Figure 4B**). Combined, these results indicate that capsule and SLO expression potentially block uptake of planktonic bacteria into respiratory epithelial cells but have less effect on biofilm bacteria. Once inside the cells their persistence is similar with planktonic bacteria being eliminated more rapidly than biofilm bacteria. Lack of both capsule and SLO act in an additive or synergistic way to increase uptake of planktonic bacteria and result in prolonged intracellular persistence.

### Role of other virulence factors during GAS association, internalization and persistence

The cysteine protease SpeB and the toxin SLS are thought to play an inhibitory role during uptake of planktonic GAS bacteria into epithelial cells (Jadoun et al., 2002; Sierig et al., 2003). Downregulation of SpeB and SLS have been observed in biofilm bacteria and their expression inhibit biofilm formation, however their role during uptake of biofilm bacteria into respiratory epithelial cells has not been elucidated (Marks et al., 2014; Alamiri et al., 2020).

The role of these factors during association, internalization, and persistence was investigated using isogenic mutants of GAS-771 (M3) lacking SpeB (*ΔspeB*) or SLS (*ΔsagA*) (**Figure 4** and **Figure S4**). Presence or absence of SpeB or SLS in planktonic bacteria did not significantly affect the cell association levels when compared to their corresponding WT strain (**Figure S4**, green). Furthermore, no significant difference in internalization efficiency (**Figure 4A**) or total numbers (**Figure S4**) were detected for either mutant compared to their corresponding WT strain, however, a more rapid elimination of the Δ*sagA* mutant (eliminated at 7 h) was observed (**Figure 4B**).

Although cells were inoculated with a higher MOI of planktonic bacteria (MOI = 1) than biofilm bacteria (MOI = 0.1), biofilm bacteria of both mutants still internalized in significantly higher numbers into respiratory epithelial cells (**Figure S4**), This was, however, not reflected in comparably increased internalization efficiencies (**Figure 4A**). Similarly, even though different MOIs were used for inoculation of the wild-type and the mutant strains, similar internalization efficiencies and total numbers were detected. On the other hand, the SpeB-negative mutant showed a reduced persistence level (eliminated at 44 h) as compared to its WT strain (>44 h) (**Figure 4B**). These results show no major role of SpeB and SLS during uptake of planktonic bacteria but indicate that they may play a role during uptake of biofilm bacteria. SpeB mediates prolonged intracellular survival of biofilm bacteria whereas SLS affects the survival of internalized planktonic bacteria only.

### Localization of GAS planktonic and biofilm bacteria inside epithelial cells

**Figure 5:**
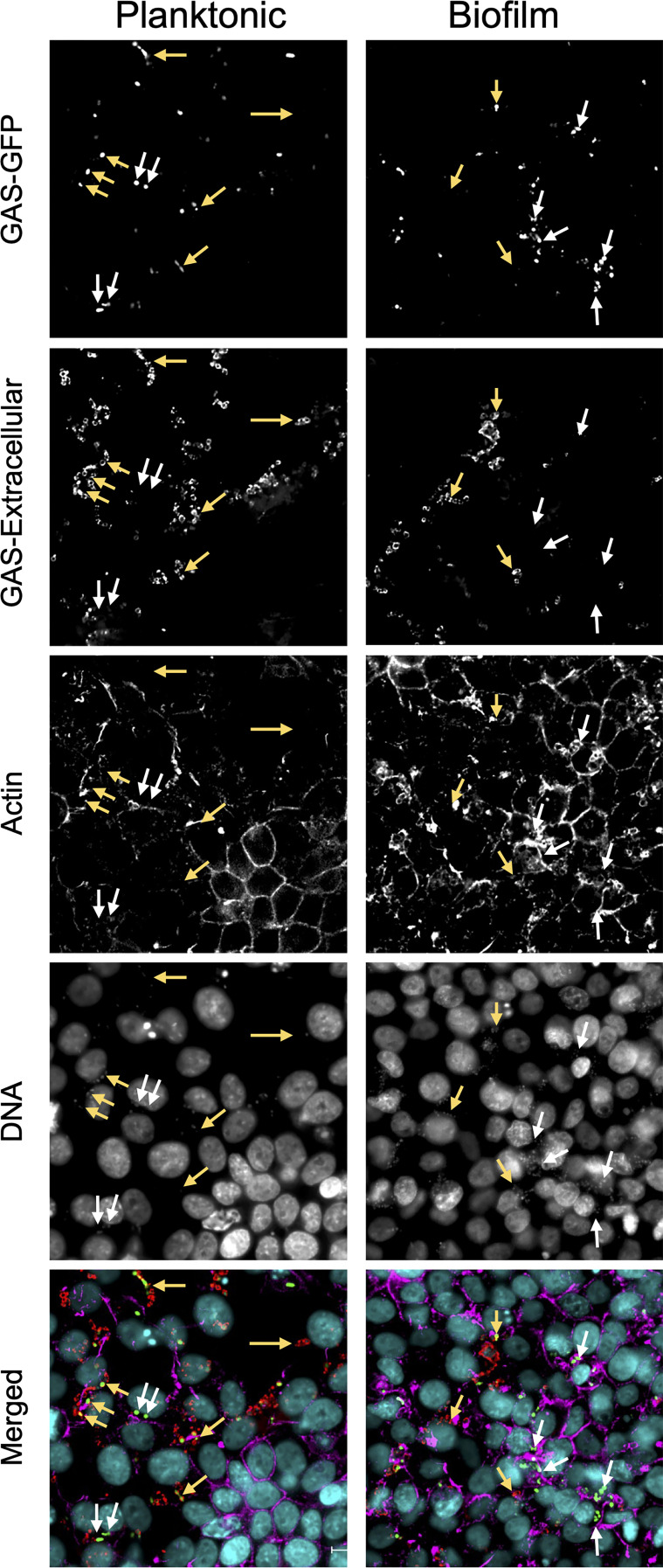
Co-localization of intracellular non-encapsulated GAS bacteria in respiratory epithelial cells. To localize intracellular planktonic or biofilm bacteria in GAS, a GFP-expressing M3 (GAS-771) strain lacking capsule (Δ*hasA*-GFP) was grown planktonically or as a biofilm and used to infect live respiratory epithelial H292 cells at an MOI of 10 for 4 h and then fixed with 4% PFA. To visualize cell structures, samples were stained with Hoechst (DNA; blue) and AlexaFluor 561 conjugated phalloidin (actin; orange). Extracellular bacteria were stained with an anti-GAS antibody and counterstained with anti-goat antibody conjugated with AlexaFluor 647 (GAS; red) and indicated using yellow arrows whereas intracellular bacteria (green only) are indicated using white arrows. Fluorescence was visualized using a Nikon Ti2 Eclipse microscope and NIS-Elements software. Images are representative 60X magnification images from multiple locations selected from a 3x3 area imaged at 20X magnification for each sample. Z-stacks were collected in each instance, and deconvolved center planes are shown. Size bar = 10 µm.

Previous studies have indicated that planktonic GAS are internalized alone or as larger aggregates into epithelial cells and associated with either lysosomes, autophagosomes, or an uncharacterized intracellular environment, depending on strains and cell types (Nakagawa et al., 2004; Hakansson et al., 2005; O’Seaghdha and Wessels, 2013). SLO has been shown to divert intracellular trafficking and direct cells to environments where they survive better (Hakansson et al., 2005; O’Seaghdha and Wessels, 2013). This was verified here when SLO was absent together with capsule but not when absent alone (**Figure 4**). To visualize internalization of planktonic and biofilm bacteria, respiratory epithelial cells were inoculated with GFP-expressing GAS for 4 hours, washed and fixed in 4% paraformaldehyde. Extracellular bacteria were stained with anti-GAS antibody and the cells were counterstained for actin, and bacterial and nuclear DNA were stained with DAPI (**Figure 5**). Capsule-negative GAS-771 (Δ*hasA*; strain 188) and its SLO-negative variant (Δ*hasA*Δ*slo*; strain 781) were used as these strains have been used successfully in previous studies investigating intracellular trafficking of planktonic GAS (Hakansson et al., 2005).

As expected, uptake of planktonic GAS-188 resulted in internalization of individual bacteria located as indicated by being present at a depth plane inside the cell (**Figure 5**; white arrows). After inspection of numerous sections, most bacteria remained extracellular, indicated in this representative image by yellow arrows. Similar to previous studies (Hakansson et al., 2005) and internalization assays above (**Figure S4**), 781 (Δ*hasA*Δ*slo*) planktonic bacteria that lack SLO were internalized in significantly higher numbers and were more often found localized in the perinuclear region of the cytoplasm and resulted in a higher co-localization with actin filaments (**Figure S5**). Localization of biofilm bacteria of both GAS strains showed a pattern similar to 781 (Δ*hasA*Δ*slo*) planktonic bacteria with higher level of bacterial internalization localized in the perinuclear region of the cytoplasm (**Figure 5** and **Figure S5**).

These studies indicate that planktonic GAS that are internalized into epithelial cells show differences in both intracellular numbers and localization than the corresponding biofilm bacteria and that SLO, at least in part, is responsible for this pattern as both planktonic and biofilm bacteria lacking SLO show the same perinuclear localization pattern. However, the specific uptake mechanism or intracellular localization of the bacteria cannot be elucidated from these experiments.

### Cellular uptake pathways utilized by GAS bacteria during internalization

Internalization of planktonic GAS bacteria require actin rearrangement and is thought to involve binding of GAS to host cell integrins directly or indirectly by using fibronectin or other plasma proteins as bridging molecules (Cue et al., 1998; Ozeri et al., 1998). Internalization into epithelial cells has also been suggested to be mediated through clathrin-mediated uptake (Logsdon et al., 2011). However, the cellular uptake pathway utilized by biofilm bacteria is not known.

To address this, live epithelial cells were pre-treated for 1 h with a set of inhibitors targeting proteins or lipids involved in general cell internalization pathways, such as actin (cytochalasin D), microtubulin (nocodazole) or dynamin (dynasore), or involved in more specific pathways involving clathrin (pitstop 2) or cholesterol in lipid-rafts (nystatin). We also used the micropinocytosis inhibitor wortmannin (PI3 Kinase inhibitor) that also have been shown to have roles in intracellular trafficking. The inhibitory activity of each inhibitor was confirmed by visualizing uptake of fluorescently labeled transferrin that uses clathrin-mediated endocytosis to enter cells, a process that also requires actin and dynamin (Mayle et al., 2012), fluorescent cholera toxin subunit B that uses primarily lipid-raft mediated endocytosis, an actin-dependent process (Hansen et al., 2005), and fluorescently labeled high molecular weight dextran (70,000 MW) that uses macropinocytosis, a process that also requires cytoskeleton rearrangement (Logsdon et al., 2011), (**Figure S6**).

**Figure 6:**
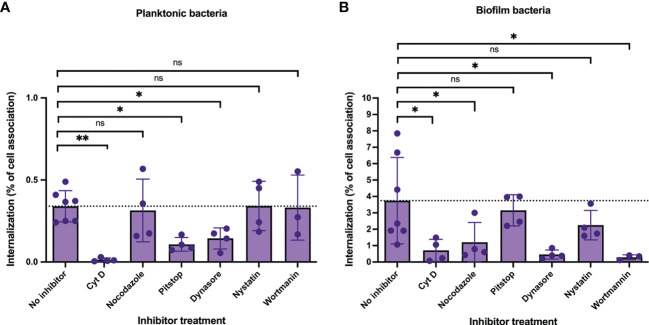
Cellular uptake pathways utilized by intracellular GAS bacteria in respiratory epithelial cells. To determine the cellular uptake pathways utilized during GAS internalization, live respiratory epithelial (H292) cells were pre-treated with inhibitors targeting proteins involved in uptake pathways, including cytochalasin D (actin, 50 μg/ml), nocodazole (microtubulin, 10 μg/ml), pitstop 2 (clathrin-mediated uptake, 11.8 μg/ml), dynasore (dynamin, 25.7 μg/ml), nystatin (lipid-raft mediated uptake, 7.5 μg/ml) or wortmannin (macropinocytosis, 100 µg/ml) for 1h. Inhibitor- or non-treated cells (No inhibitor) were inoculated with planktonic **(A)** or biofilm **(B)** *S. pyogenes* GAS-771 in RPMI supplemented with 2% serum (please observe the different scales of the Y-axis). Cells were inoculated with bacteria at 34°C for 4 h without antibiotic treatment, or 2.5 h followed by antibiotic treatment for 1.5 h to eliminate extracellular bacteria. Bacterial internalization and cell-association levels were assessed by determining the Log_10_ CFUs, 4 h post infection (data shown in **Figure S4**). Internalization was then presented in the graph as the percentage of cell-associated bacteria that were internalized for each experiment (purple). The dotted line represents the internalization level for the no inhibitor control (0.34% for planktonic organisms and 3.74% for biofilm bacteria). One-way ANOVA using Dunnett’s multiple comparison tests were used to compare the means of inhibitor treated cells as compared to the non-treated cells ± SD (n = 3 to 7 experiments). For the internalization experiments each individual significance level is displayed in the figure. For all statistical analyses *P < 0.05, **P < 0.01, and ns, non-significant difference. .

Next, cells were pre-incubated with inhibitors followed by inoculation with GAS-771 planktonic and biofilm bacteria. After 4 hours, cell internalization efficiencies as percentage of total cell-association (**Figure 6**) and total internalization numbers (**Figure S7**, purple bars) were determined. In parallel, bacterial CFUs in the cell supernatants and lysates were investigated to exclude any effect of inhibitors on bacterial growth and/or adherence. None of the inhibitors affected bacterial growth or significantly affected total bacterial cell association (**Figure S7**, green bars). Inhibition of actin polymerization (Cyt D) and dynamin (Dynasore) significantly reduced the bacterial internalization efficiencies of both planktonic and biofilm bacteria (**Figure 6**). In agreement with previous results, inhibition of clathrin-mediated uptake (Pitstop) reduced the internalization efficiency of planktonic bacteria (**Figure 6A**), however uptake of biofilm bacteria was not significantly affected (**Figure 6B**). Inhibitors targeting microtubulin (nocodazole) and macropinocytosis (wortmannin) significantly reduced the internalization of biofilm bacteria (**Figure 6B**) but did not affect the uptake of planktonic bacteria (**Figure 6A**, purple bars). Inhibition of lipid-raft mediated uptake (nystatin) did not cause any significant inhibition on the uptake of either planktonic or biofilm bacteria (**Figure 6**). The data with all inhibitors showed the same results also when total internalization numbers were compared (**Figure S7**).

These results confirm previous studies and show that uptake of planktonic bacteria involves both general (actin, dynamin) and specific uptake pathways (clathrin-mediated uptake) in respiratory epithelial cells. On the other hand, uptake of biofilm bacteria was not affected by inhibition of clathrin-mediated uptake, but was reduced after inhibition with wortmannin, suggesting the involvement of other mechanisms, potentially macropinocytosis, that also rely on general uptake proteins, such as actin and dynamin.

## Discussion

*Streptococcus pyogenes* is the leading cause of pharyngeal infections in children. Additionally, the prevalence of respiratory infections, such as pneumonia or otitis media (OM), due to GAS are increasing (Carapetis et al., 2005; Tan et al., 2014; Efstratiou and Lamagni, 2022). GAS colonization of keratinocytes (skin and oropharynx) or respiratory epithelial cells (nasopharynx) are common and always precedes an infectious episode (Muotiala et al., 1997; Brouwer et al., 2016). The treatment of choice for such infections is penicillin or amoxicillin, which kills GAS bacteria residing outside of cells (Vannice et al., 2020). However, GAS are known to hide within cells and recurrent pharyngeal infections, often with the same strain, are common, as they re-emerge upon completed antibiotic treatment (Ogawa et al., 2011).

Adherence and internalization of GAS into epithelial cells have been previously studied using broth-grown, planktonic bacteria (Schrager et al., 1996; Hakansson et al., 2005; Logsdon et al., 2011; Marks et al., 2014). However, less information is available for biofilm bacteria. Upon colonization of epithelial cells, GAS forms biofilms and initial studies with biofilm bacteria have shown that they internalize into keratinocytes at higher levels than planktonic bacteria (Marks et al., 2014). Results in this study confirm our previous findings and show that biofilm bacteria from most GAS strains internalize in higher numbers and with higher efficiency (i.e percentage of cell-associated bacteria that were found viable in the intracellular environment) than planktonic bacteria and persist inside cells for a longer time than planktonic bacteria, despite similar cell association numbers of both bacterial phenotypes and slower bacterial extracellular growth of biofilm bacteria. Also, intracellular persistence was not a result of higher initial numbers as titrating biofilm bacteria down to inocula that resulted in similar internalization as planktonic bacteria still resulted in superior survival over time. The improved internalization and persistence of biofilm bacteria are likely related to the bacterial phenotype and the variable expression of virulence factors, discussed below.

To better understand the general nature of GAS uptake in epithelial cells, multiple clinical isolates were used. GAS uptake into respiratory epithelial cells was observed by all serotypes tested, irrespective of tissue tropism. Historically, certain M-types are correlated with skin infections and others with pharyngitis and upper respiratory disease (Cunningham, 2000). More recent work has grouped the M-types based on genetic analysis and clustered them in groups A-C, which are preferentially isolated from pharyngitis patients, group D most often associated with skin infections and group E, called “generalists” isolated equally from all types of infection (McMillan et al., 2013; Sanderson-Smith et al., 2014; Bessen, 2016). Previous studies have not observed differences in initial adherence to either keratinocytes or pharyngeal cells between M-types (Loh et al., 2017), but no further interaction studies were done. Similar data was observed in this study, where cell association was not directly related to M-type grouping. Interestingly, the total numbers of internalization between planktonic organisms of the isolates used varied more substantially than the uptake of their corresponding biofilms, suggesting that the biofilm phenotype was more closely related between isolates. However, when investigating the internalization efficiencies as the percentage of cell-association found intracellularly, the internalization efficiencies of planktonic bacteria were generally low (with the exception of the M6 strain), whereas the internalization efficiency of biofilm bacteria was variable but always higher than their planktonic counterpart, except for serotype M77. Like the M3 isolate previously tested, biofilm bacteria of the M1T1, M12, M18 (all belonging to group A-C, pharyngitis-associated M-types) persisted and survived longer inside infected cells. On the other hand, for the M6 (A-C group M-type) and M11, M22, M89 or M77 (all group E M-types) strains, planktonic and biofilm bacteria persisted equally well inside the epithelial cells. Still, the internalization and persistence levels of biofilm bacteria were more similar between strains than for planktonic cells, suggesting a more homogenous biofilm phenotype.

The specific strain-associated factors that are correlated with these differences are not fully clear. To better understand this, we next investigated the role of various common virulence determinants for internalization and persistence of planktonic and biofilm bacteria inside epithelial cells. The M protein *per se* is a known adherence factor and in some M types (such as M1 and M3), the protein is required for optimal internalization of GAS into epithelial and endothelial cells (Courtney et al., 1997; Eyal et al., 2003; Ochel et al., 2014; Brouwer et al., 2016). The role of the M protein during uptake of biofilm bacteria have not been investigated previously. Using isogenic WT and M protein negative strains, we found that M1 or M5 expression did not affect either total cell association or internalization and had no major effect on persistence of the bacteria in the intracellular environment. Meanwhile, in accordance with earlier findings (Eyal et al., 2003), absence of the M3 protein reduced the level of total internalization numbers and internalization efficiency, compared with the WT strain, which was accompanied by a more rapid elimination of both planktonic and biofilm bacteria. It is, however, important to note that M proteins interact and bind to various molecules in the host environment, such as fibrinogen, fibronectin, and immunoglobulins, and that these local interactions within the host modulates M protein function and GAS virulence (Cue et al., 1998; Carlsson et al., 2005; Waldemarsson et al., 2009; Nordenfelt et al., 2012; Lood et al., 2015; Happonen et al., 2019). Thus, future studies including host factors, such as mucosal proteins and antibody, as well as mutants in bacterial adhesins besides M protein, such as fibronectin-binding proteins Protein F1 or SfbI (Molinari et al., 1997; Ozeri et al., 1998) will be of interest as they may well affect processes such as adherence and internalization.

The hyaluronic acid capsule is known to block uptake of planktonic GAS bacteria into epithelial cells (Hakansson et al., 2005). This was supported in this study as serotypes expressing low (M6 and M1T1), medium (M3) and high (M18) levels of capsule when grown in broth displayed an inverse (high, medium and low) bacterial uptake. The increased uptake of biofilm bacteria observed for encapsulated strains may well be associated with the known down-regulation of capsule gene expression in biofilm bacteria (Marks et al., 2014; Alamiri et al., 2020). This was also shown here as all strains, irrespective of capsule expression in broth, were internalized at very similar numbers and efficiency when grown as biofilms and persisted for up to or beyond the 44 h time point in the experiments. The role of capsule was investigated using an isogenic mutant of the M3 (GAS-771) strain lacking hyaluronic acid capsule. This mutant was internalized at similar numbers after a significantly lower inoculum than the WT strain, suggesting that capsule inhibited internalization. Like the unencapsulated M22 strain used in some of our experiments, an increased intracellular elimination rate was observed in the complete absence of capsule for both planktonic and biofilm bacteria. Whether capsule redirects the bacteria to a different localization within cells or contributes to an increased ability to survive in the intracellular environment is not yet clear. These findings suggest that down-regulation of capsule plays an important role in increasing the uptake of GAS biofilms, and also that capsule expression is required once biofilm bacteria enter cells to maintain prolonged persistence. Understanding this regulation will be an interesting future avenue of research.

Like capsule, the pore-forming toxin SLO have been shown to play a role during GAS internalization into epithelial cells. Planktonic bacteria secreting SLO can inhibit phagocytosis extracellularly but also intracellularly by escaping lysosomal killing and maintaining prolonged intracellular survival (Bricker et al., 2002; Hakansson et al., 2005; O’Seaghdha and Wessels, 2013). Some serotypes, with low expression of capsule and SLO, have been proposed to be eliminated intracellularly through autophagy (Nakagawa et al., 2004). Bacterial factors, such as SLO appear crucial for the fate of the bacteria as expression of SLO mediate targeting of GAS to autophagosomes but inhibit autophagolysosome fusion and bacterial death (O’Seaghdha and Wessels, 2013). The SLO-negative mutant of the M3 (GAS-771) was internalized at similar numbers, but a significantly higher efficiency, compared with the WT strain after incubation with a significantly lower inoculum, suggesting that SLO also inhibited internalization. The elimination rate of the bacteria were, however, not affected by the absence of SLO in biofilm bacteria, suggesting that for these bacteria SLO played little to no role in persistence once inside the cells, suggesting a minor role for autophagosomal trafficking. SLO was also shown to have synergistic effects with capsule. We have previously shown that a mutant lacking both capsule and SLO in the M3 (GAS-771) strain was internalized in high numbers and could be detected in lysosomes whereas its corresponding WT strain was absent in this intracellular compartment (Hakansson et al., 2005). In this study, this double mutant internalized in both higher numbers and with higher efficiency for both bacterial phenotypes compared with wild-type bacteria and showed a prolonged persistence (44 h) of planktonic bacteria when compared to its WT strain (20 h). This study shows that SLO and capsule blocks internalization of GAS bacteria in respiratory epithelial cells and that capsule have an important role in intracellular survival of biofilm bacteria.

Additionally, the cysteine protease SpeB or the toxin SLS have been shown to decrease GAS internalization into epithelial cells to various degrees (Jadoun et al., 2002; Sierig et al., 2003). Here, expression of SLS or SpeB, did not show a significant impact on either cell association or uptake of GAS into respiratory epithelial cells. A lower MOI was used for inoculation of the biofilm bacteria than the planktonic bacteria, again indicating the superior ability of biofilm bacteria to internalize into epithelial cells. Results obtained here show that SLS plays a minor role during persistence of planktonic bacteria, whereas SpeB is needed to maintain prolonged survival of biofilm bacteria. In previous studies using planktonic M1T1 GAS, SpeB was reported to use a proteolytic mechanism to avoid ubiquitinylation and recognition of the autophagosomal marker LC3 and related adaptor proteins to avoid autophagy (Barnett et al., 2013). No effect was observed for survival of planktonic organisms in this study. However, whether this mechanism is involved in persistence of biofilm bacteria will be interested to study in the future.

The results with bacterial uptake and persistence suggest that biofilm bacteria are internalized in higher numbers and persist for longer periods of time intracellularly. This may indicate that the bacteria are internalized through different internalization pathways, that they are trafficked to different intracellular environments and/or that the biofilm phenotype may provide better protection against antimicrobial agents in the intracellular environment. Planktonic bacteria of GAS-771 and other serotypes have been shown to be internalized into epithelial cells through a clathrin-mediated pathway, and further trafficked to autophagosomes and lysosomes, a pathway that is perturbed by the expression of SLO and SLS (Nakagawa et al., 2004; Hakansson et al., 2005; Logsdon et al., 2011; O’Seaghdha and Wessels, 2013). There are also studies suggesting that some serotypes of GAS can be released from endosomes and replicate in the cytoplasm of epithelial cells and macrophages (Barnett et al., 2013; O’Neill et al., 2016). To study intracellular persistence and survival of GAS, gentamicin is primarily used to eliminate extracellular bacteria (Barnett et al., 2013; O’Neill et al., 2016; Nozawa et al., 2021). However, under certain conditions, such as the short antibiotic exposure times used in this study, our previous studies (Hakansson et al., 2005; Logsdon et al., 2011; O’Seaghdha and Wessels, 2013) and those of other groups (LaPenta et al., 1994; Nakagawa et al., 2004) have shown that gentamicin alone fail to completely eliminate extracellular bacteria and addition of a low concentration of penicillin is required. A recent study by Barnett et al. found that the addition of penicillin, that rapidly enters the cytoplasm but not the endosomal compartment of cells could inhibit bacterial cytosolic growth (Barnett et al., 2013). Therefore, when interpreting our data, it should be noted that although we have controlled for the complete elimination of extracellular bacteria, it cannot be ruled out that the addition of penicillin could also have some effect on the survival of intracellular bacteria as has been shown under some experimental conditions.

Whether biofilm bacteria use the same uptake pathway or intracellular trafficking as planktonic bacteria is unknown. Inhibition of general internalization mechanisms, such as actin polymerization and dynamin function significantly inhibited internalization of both planktonic and biofilm bacteria. In agreement with earlier studies, uptake of planktonic bacteria into epithelial cells most likely involved clathrin, based on inhibition with Pitstop, which was not seen for biofilm bacteria. Instead, inhibitors mainly targeting tubulin assembly and macropinocytosis blocked uptake of biofilm bacteria. These internalization results, showing different inhibition profiles, suggest the involvement of different cell internalization pathways for planktonic and biofilm bacteria into epithelial cells. The inhibitor experiments also suggest that biofilm aggregates may be taken up through macropinocytosis. However, as most inhibitors have off-target effects that may influence other internalization pathways than those targeted (Rennick et al., 2021), the specific uptake mechanism involved need to be confirmed using other techniques, which will be done in future studies. The increased persistence of biofilm bacteria also suggests differences in intracellular trafficking, where biofilm bacteria were able to localize in environments where they could better survive. As penicillin, a common antibiotic used for treatment of GAS infection, is taken up in the cytosol of some cells, this indicates that biofilm bacteria potentially elude the cytosolic environment and further suggest that biofilm bacteria could constitute the bacterial phenotype associated with recurring infection after antibiotic treatment.

Visualization of bacterial uptake by microscopy supported this difference. Planktonic bacteria were internalized and found alone or few together in the intracellular compartment. At the same time longer chains of extracellular bacteria were observed. Biofilm bacteria were found in aggregates inside of bacteria, localized in the perinuclear area. This pattern was similar to the morphology of the uptake of SLO-negative bacteria, that tend to traffic to lysosomes or autophagosomes (Hakansson et al., 2005) and accumulate in the perinuclear region. Quantification of relative numbers and relationship to size and shape over time would likely yield interesting insights into the mechanisms of internalization and subsequent fate. However, this would require further development in image analysis algorithms and establishing suitable live imaging conditions. Although the morphology was similar, further studies using specific markers for relevant intracellular environments are required to understand the specific localization of the bacterial phenotypes. Uptake of a group of bacteria rather than single bacteria in invasomes using actin, talin-1 and integrin-β1 have been observed in *Bartonella henselae*, and it could be of potential interest to see if GAS biofilms use the same pathway using inhibitors targeting talin-1 and integrin-β1 (Eicher and Dehio, 2012).

In conclusion, results presented in this study have the potential to help in better understanding respiratory infections caused by GAS and the mechanisms used by these bacteria to escape antibiotic treatment. Studying the role of virulence factors during GAS pathogenesis will also help in defining therapeutic targets during treatment of GAS infections.

## Data availability statement

The raw data supporting the conclusions of this article will be made available by the authors, without undue reservation.

## Author contributions

FA, OA, and SD designed and performed the experiments of the study as well as analyzed the results and produced graphs and figures for the manuscript. FA, PN and AH designed the study, analyzed and compiled the data, and wrote the manuscript. All authors contributed to the article and approved the submitted version.
