# Supplementary material for: Role of serotype and virulence determinants of Streptococcus pyogenes biofilm bacteria in internalization and persistence in epithelial cells in vitro
Source: Front Cell Infect Microbiol. 2023 May 10;13:1146431. doi: 10.3389/fcimb.2023.1146431 (PMC10206268; doi:10.3389/fcimb.2023.1146431)
Supplement: Supplementary file 1 [file DataSheet_1.pdf]

SUPPLEMENTAL INFORMATION

Role of serotype and virulence determinants of *Streptococcus pyogenes* biofilm bacteria in internalization and persistence inside epithelial cells *in vitro*

By: Feiruz Alamiri, Oscar André, Supradipta De, Pontus Nordenfelt and Anders P. Hakansson

SUPPLEMENTARY FIGURES

Figure S1

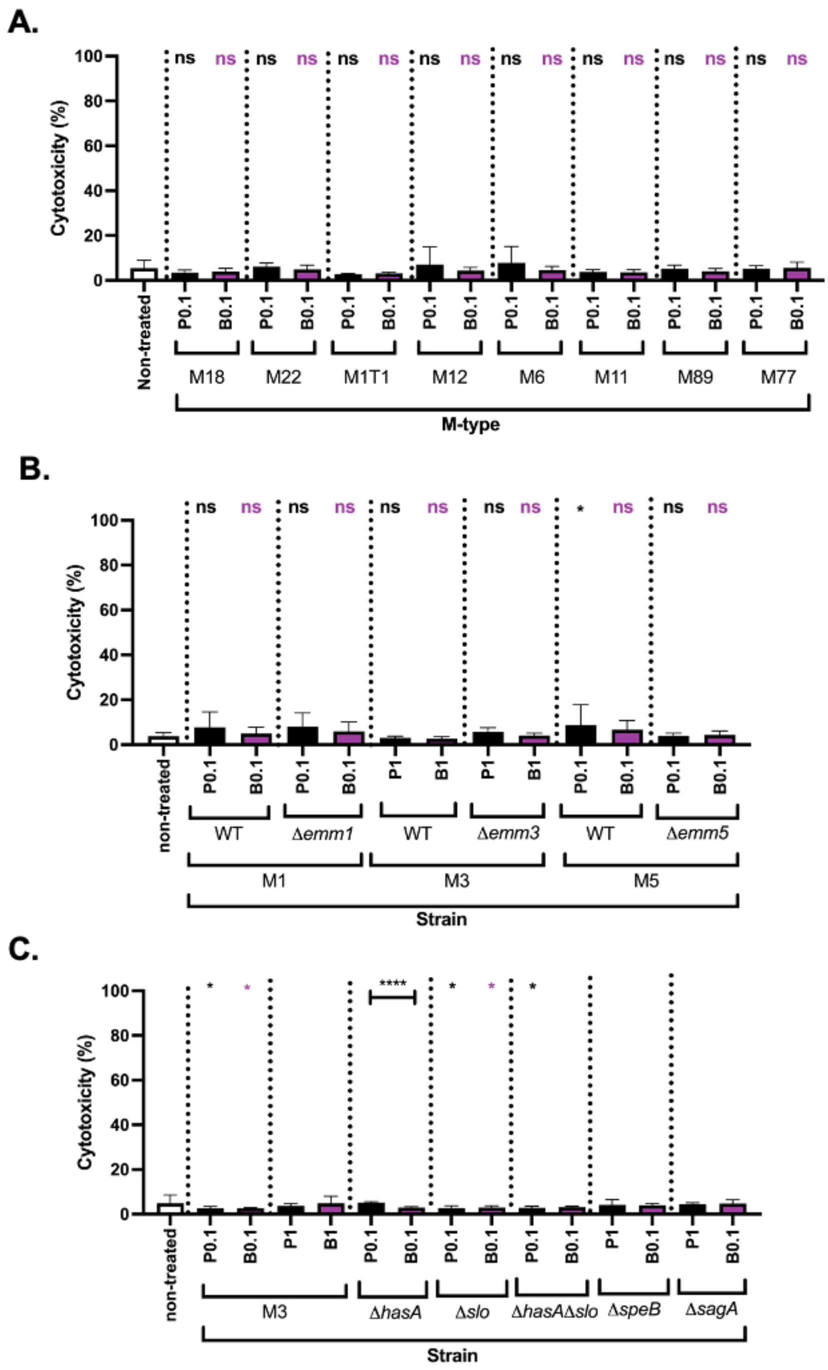

Figure S1. Cellular viability of live respiratory cells infected with GAS planktonic and biofilm bacteria. Live respiratory epithelial (H292) cells were infected with different serotypes of *S. pyogenes* grown planktonically or as biofilms. Infected cells were incubated at 34°C for 4 h without antibiotic treatment, or 2.5 h followed by antibiotic treatment with gentamicin (500 μg/ml) and penicillin (20 μg/ml) for 1.5 h to eliminate extracellular bacteria. To assess the cell viability, the cytotoxicity percentage of non-treated cells (white bar) or cells infected with planktonic (black bar) or biofilm bacteria (purple bar), was determined by measuring the lactate dehydrogenase (LDH) release into culture supernatant during antibiotic treatment 4 h post infection. (**A**) To determine the role of M protein on cell viability, the viability of epithelial cells was determined after exposure to GAS M1T1, M6, M11, M12, M18, M22, M77 or M89 for 4 hours. (**B**) To assess the role of M protein during GAS infection, M1 (SF370), M3 (GAS-771) and M5 (Manfredo) strains expressing wild-type (WT) or lacking the M protein (Δ*emm*) were used to infect live respiratory epithelial cells as indicated above. (**C**) To determine the role of virulence factors during GAS infection, M3 (GAS-771) strain expressing wild-type (WT) or lacking capsule (Δ*hasA*), SLO (Δ*slo*), or both (Δ*hasA*Δ*slo*), or SpeB (Δ*speB*), or SLS (Δ*sagA*), were used to infect live epithelial cells as indicated above. P1 (or P0.1) and B1 (or B0.1) represent planktonic (P) and biofilm (B) bacteria inoculated at a multiplicity of infection (MOI) of 1 (or 0.1). The significant difference in LDH release from cells treated with planktonic bacteria (black), or biofilm bacteria (purple), as compared to each other is shown with a black line, or compared to the non-treated samples is shown on top of each bar. One-way ANOVA using Dunnett's multiple comparison tests were used to compare the viability of GAS treated cells with non-treated and results are presented as mean ± SD for (**A**) n = 12, except for M11 where n = 9; (**B**) n = 12, except for Δ*emm3* where n = 9, or non-treated or M3WT where n = 15; (**C**) n = 12, except for Δ*hasA*, Δ*SpeB*, or Δ*sagA* where n = 9, or non-treated or M3WT where n = 21. For all statistical analyses *, $P < 0.05$, ****, $P < 0.0001$, and ns, non-significant difference.

Figure S2

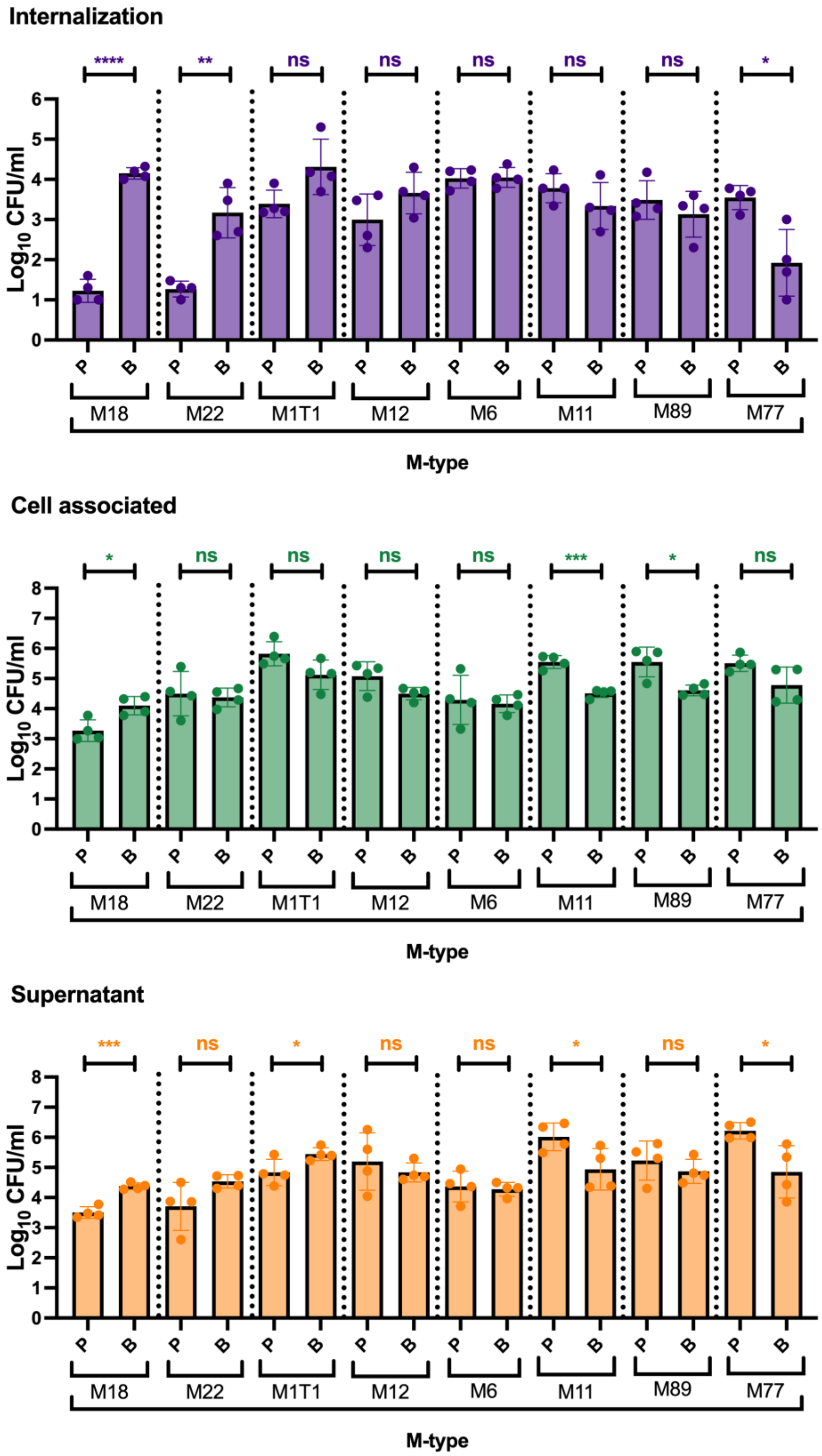
Internalization
**** ** ns ns ns ns ns *
Log10 CFU/ml
P B P B P B P B P B P B P B P B
M18 M22 M1T1 M12 M6 M11 M89 M77
M-type
Cell associated
* ns ns ns ns *** * ns
Log10 CFU/ml
P B P B P B P B P B P B P B P B
M18 M22 M1T1 M12 M6 M11 M89 M77
M-type
Supernatant
*** ns * ns ns * ns *
Log10 CFU/ml
P B P B P B P B P B P B P B P B
M18 M22 M1T1 M12 M6 M11 M89 M77
M-type

Figure S2. Bacterial growth and cell association, of different GAS serotypes in respiratory epithelial cells. Live respiratory epithelial (H292) cells were infected with *S. pyogenes* M1T1, M6, M11, M12, M18, M22, M77 or M89 grown planktonically or as biofilms. Cells were inoculated with bacteria at 34°C for 2.5 h followed by antibiotic treatment for 1.5 h to eliminate extracellular bacteria or for 4 h without antibiotic treatment to assess total cell-association and growth in the culture supernatant. Bacterial internalization (Internalization, purple bars), bacterial total association to the cells (Association, green bars) or growth in the culture supernatant (Supernatant, orange bars) were assessed by determining the Log_{10} CFUs, 4 h post infection. P and B indicate inoculation of bacteria with planktonic (P) or biofilm (B) bacteria at a multiplicity of infection (MOI) of 0.1. The results represent mean data from four separate experiments ± SD (n = 4) with individual data points presented in the graph. Differences in internalization (purple), association (orange), or supernatant growth (orange), between planktonic and biofilm bacteria for each M type was compared using one-way ANOVA using Dunnett's multiple comparison test and is displayed on top of each bar. For all statistical analyses *, $P < 0.05$, **, $P < 0.01$, ***, $P < 0.001$, ****, $P < 0.0001$ and ns, non-significant difference.

Figure S3

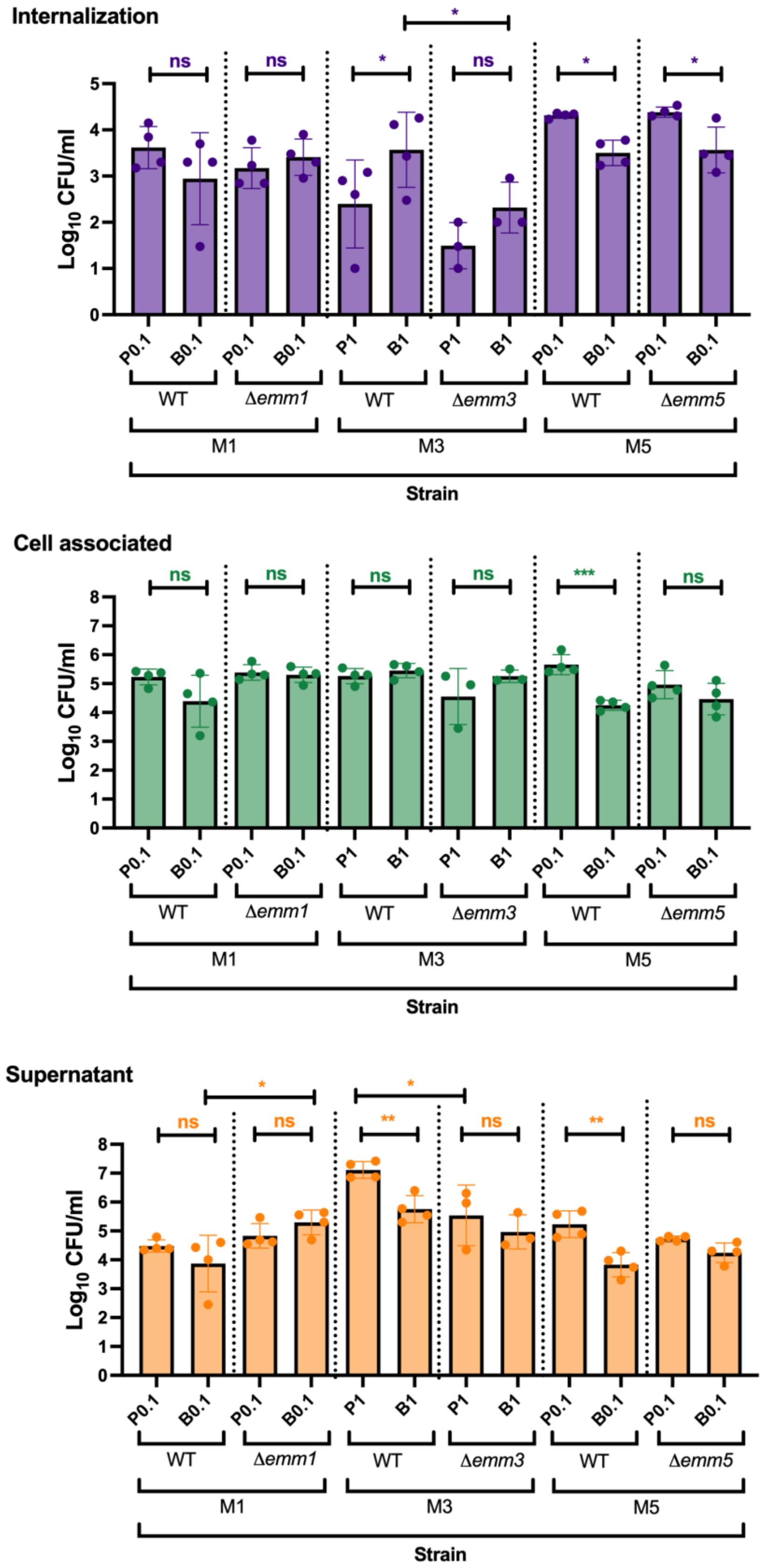
Internalization
Log10 CFU/ml
ns
ns
*
ns
*
*
*
P0.1
B0.1
P1
B1
WT
Δemm1
Δemm3
Δemm5
M1
M3
M5
Strain
Cell associated
ns
***
Supernatant
**

Figure S3. Role of the M protein in GAS bacterial growth and cell association, during respiratory epithelial cell infection. GAS M1 (SF370), M3 (GAS-771) and M5 (Manfredo) strains expressing (WT) or lacking the M protein (Δ*emm*) and grown planktonically or as biofilms were used to infect live respiratory epithelial (H292) cells. Cells were inoculated with bacteria at 34°C for 2.5 h followed by antibiotic treatment for 1.5 h to eliminate extracellular bacteria or for 4 h without antibiotic treatment to assess total cell-association and growth in the culture supernatant. Bacterial internalization (Internalization, purple bars), total bacterial association to the cells (Association, green bars) or bacterial growth in the culture supernatant (Supernatant, orange bars) were assessed by determining the Log_{10} CFUs, 4 h post infection. P1 (or P0.1) and B1 (or B0.1) represent planktonic (P) or biofilm (P) bacteria used at a multiplicity of infection (MOI) of 1 (or 0.1). The results represent mean data from four (or three in Δ*emm3*) separate experiments ± SD. Differences in internalization (purple), cell association (green) or supernatant growth (orange), between planktonic and biofilm bacteria in each strain, or between WT and Δ*emm* for each M type, was compared using one-way ANOVA using Dunnett's multiple comparison tests and is displayed by colored stars in the graph. For all statistical analyses *, $P < 0.05$, **, $P < 0.01$, ***, $P < 0.001$, ****, $P < 0.0001$ and ns, non-significant difference.

Figure S4

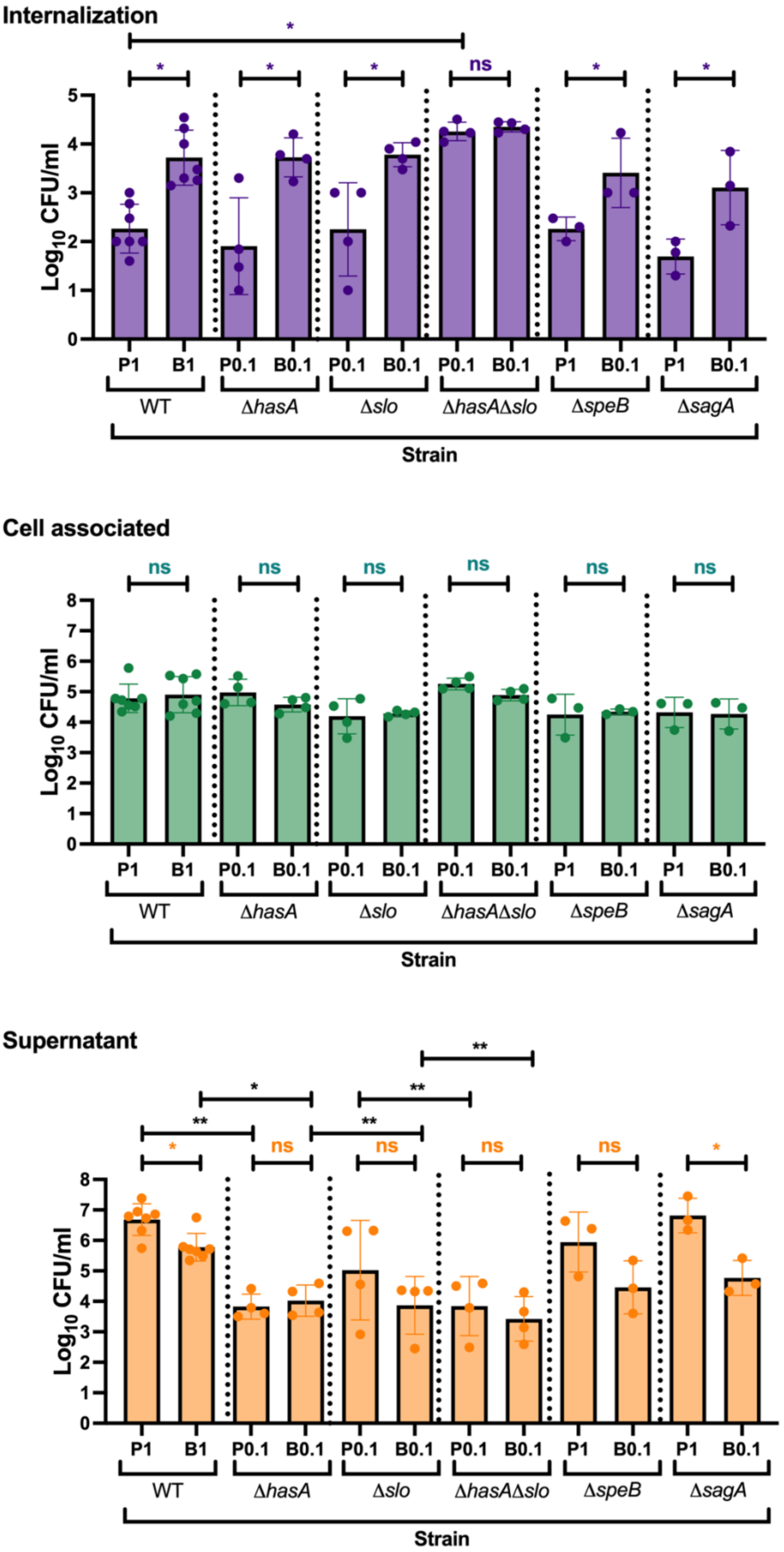
Internalization
*
* * * ns * *
Log10 CFU/ml
0 1 2 3 4 5
P1 B1 P0.1 B0.1 P0.1 B0.1 P0.1 B0.1 P1 B0.1 P1 B0.1
WT ΔhasA Δslo ΔhasAΔslo ΔspeB ΔsagA
Strain
Cell associated
ns ns ns ns ns ns
Log10 CFU/ml
0 1 2 3 4 5 6 7 8
P1 B1 P0.1 B0.1 P0.1 B0.1 P0.1 B0.1 P1 B0.1 P1 B0.1
WT ΔhasA Δslo ΔhasAΔslo ΔspeB ΔsagA
Strain
Supernatant
**
* **
** **
* ns ns ns ns *
Log10 CFU/ml
0 1 2 3 4 5 6 7 8
P1 B1 P0.1 B0.1 P0.1 B0.1 P0.1 B0.1 P1 B0.1 P1 B0.1
WT ΔhasA Δslo ΔhasAΔslo ΔspeB ΔsagA
Strain

Figure S4. Role of virulence factors in GAS bacterial growth and cell association, during respiratory epithelial cell infection. To determine the role of virulence factors during GAS infection, M3 (GAS-771) strain expressing wild-type (WT) or lacking capsule (Δ*hasA*), SLO (Δ*slo*), or both (Δ*hasA*Δ*slo*), or SpeB (Δ*speB*), or SLS (Δ*sagA*) grown planktonically or as biofilms, were used to infect live respiratory epithelial (H292) cells. (**A**) Cells were inoculated with bacteria at 34°C for 2.5 h followed by antibiotic treatment for 1.5 h to eliminate extracellular bacteria or for 4 h without antibiotic treatment to assess total cell-association and growth in the culture supernatant. Bacterial internalization (Internalization, purple bars), total bacterial association to the cells (Association, green bars) or bacterial growth in the culture supernatant (Supernatant, orange bars) were assessed by determining the Log_{10} CFUs, 4 h post infection. P1 (or P0.1) and B1 (or B0.1) represent planktonic (P) and biofilm (B) bacteria a multiplicity of infection (MOI) of 1 (or 0.1). The results represent mean data from three (in Δ*speB,* or Δ*sagA*), four (in Δ*hasA,* Δ*slo*, or Δ*hasA*Δ*slo*), or seven (M3WT), separate experiments ± SD (n = 3 experiments in Δ*speB,* or Δ*sagA*, or n = 4 experiments in Δ*hasA,* Δ*slo*, or Δ*hasA*Δ*slo,* or n = 7 experiments in M3WT). Differences in internalization (purple), association (green) or supernatant growth (orange) was compared using one-way ANOVA using Dunnett's multiple comparison tests. Differences between planktonic and biofilm bacteria for each strain is shown on top of the black line in colored stars and differences between planktonic and biofilm bacteria in WT and mutant strains and are shown on top of each bar with black stars. For all statistical analyses *, $P < 0.05$, **, $P < 0.01$, ***, $P < 0.001$, ****, $P < 0.0001$ and ns, non-significant difference.

Figure S5

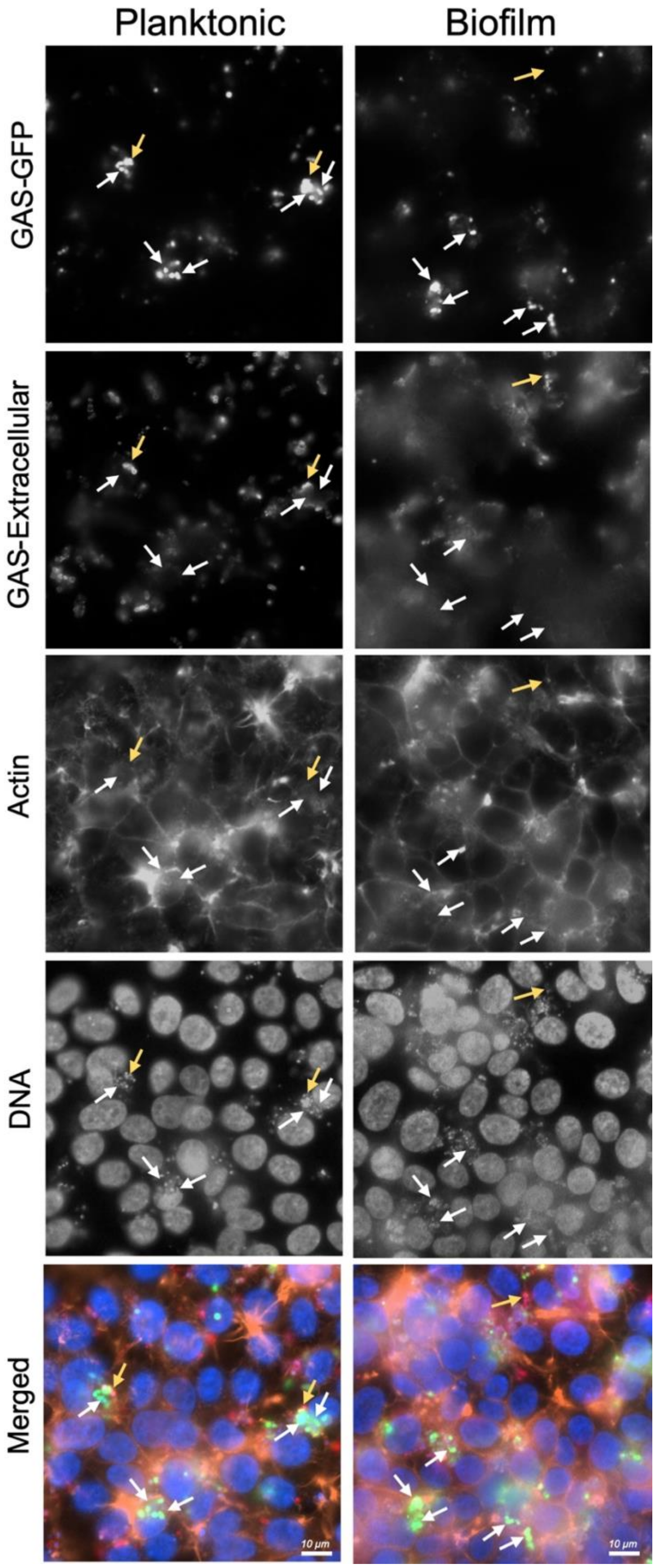
Planktonic
Biofilm
GAS-GFP
GAS-Extracellular
Actin
DNA
Merged
10 µm
10 µm

Figure S5. Co-localization of intracellular GAS bacteria lacking capsule and SLO within respiratory epithelial cells. To investigate localization of intracellular planktonic or biofilm bacteria in GAS, a GFP-tagged M3 (GAS-771) strain lacking capsule and SLO (Δ*hasA*Δ*slo* -GFP) was used to infect live respiratory epithelial cells at an MOI of 10 for 4 h and then fixed with 4% PFA. To visualize cell structures, samples were stained with Hoechst (DNA; blue) and AlexaFlour 568 conjugated phalloidin (actin; orange). Both extracellular and intracellular bacteria express GFP (GAS; green), however extracellular bacteria were labeled with an goat-anti-GAS antibody counterstained with AlexaFlour 647 conjugated donkey-anti-goat antibody (GAS; red) and indicated using yellow arrows whereas intracellular bacteria are pointed out using white arrows. Fluorescence was visualized using a Nikon Ti2 Eclipse microscope and NIS-Elements software. Images are representative 60X magnification images from multiple locations selected from a 3x3 area imaged at 20X magnification for each sample. Z-stacks were collected in each instance, and deconvolved center planes are shown. Size bar = 10 µm.

Figure S6

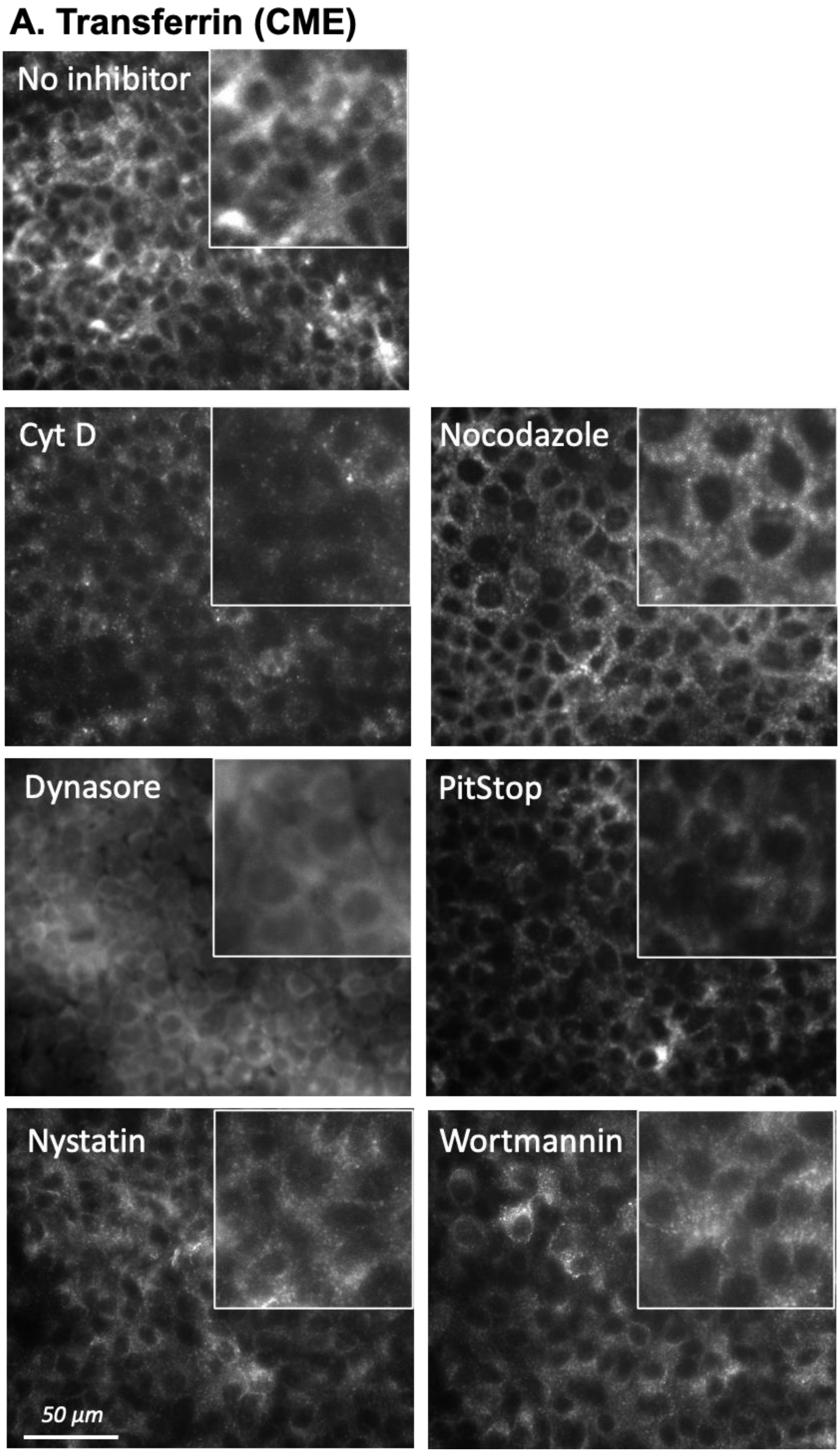
A. Transferrin (CME)
No inhibitor
Cyt D
Nocodazole
Dynasore
PitStop
Nystatin
Wortmannin
50 µm

B. Cholera toxin B (LRME)

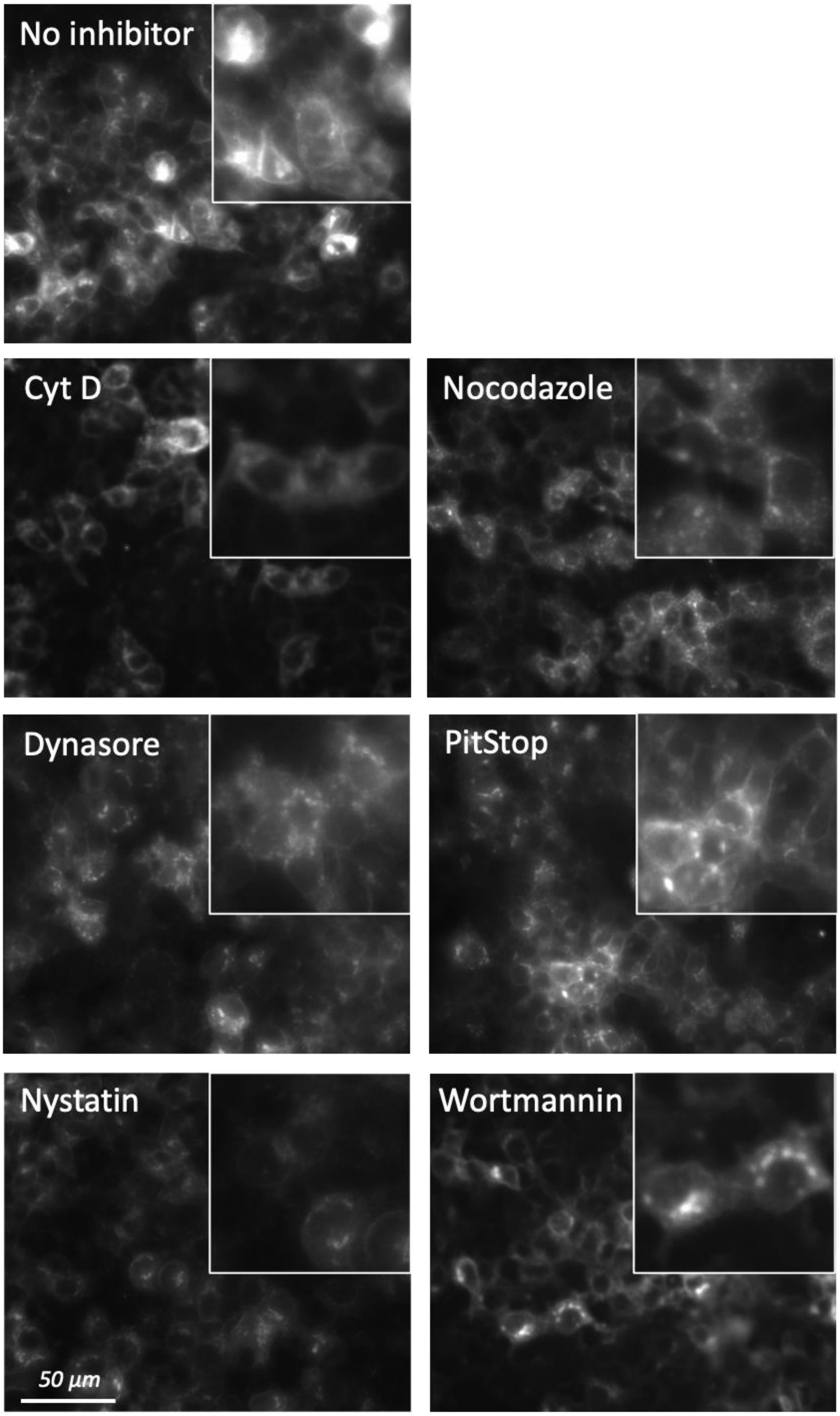

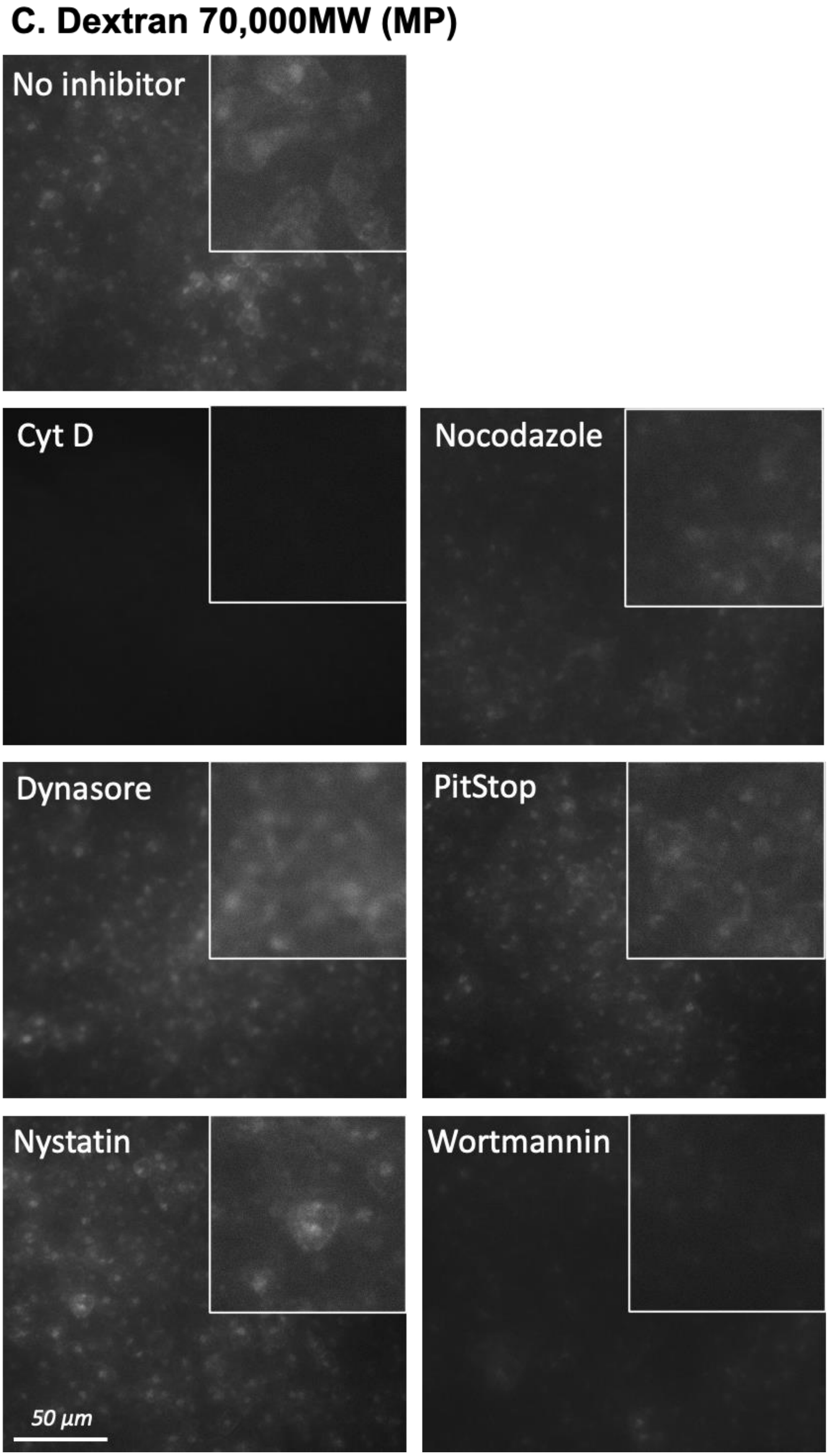
C. Dextran 70,000MW (MP)
No inhibitor
Cyt D
Nocodazole
Dynasore
PitStop
Nystatin
Wortmannin
50 µm

Figure S6. Uptake inhibition of transferrin, cholera toxin B, and dextran 70,000 MW. To confirm the effective inhibition of cellular uptake pathways using the tested inhibitor concentrations, uptake of (**A**) fluorescently labeled transferrin that uses clathrin-mediated endocytosis (CME), (**B**) cholera toxin subunit B that uses lipid-raft mediated endocytosis (LRME), and (**C**) high molecular weight dextran 70,000 MW that uses macropinocytosis (MP) was investigated in respiratory epithelial cells. Live respiratory epithelial cells were pre-incubated at 34 °C for 1 h with inhibitors targeting actin (cytochalasin D, 50 μg/ml), microtubulin (nocodazole, 10 μg/ml), dynamin (dynasore, 25.7 μg/ml) clathrin- (pitstop 2, 11.8 μg/ml), lipid-raft mediated endocytosis (nystatin, 7.5 μg/ml), or macropinocytosis (wortmannin, 100 μg/ml). Cells were then treated with 25 μg/ml of transferrin or 4 μg/ml of cholera toxin subunit B, both conjugated with AlexaFluor-488 or fluorescein-conjugated dextran 70,000 MW and further incubated for 20 min, 1 h, or 2 h respectively, at 34 °C. Fluorescence was visualized using an inverted fluorescent microscope (Zeiss Axiovert.A1, Zeiss). The scale bar represents 50 μm. Punctuate intracellular fluorescence indicate uptake of fluorescent cargo into live cells.

Figure S7

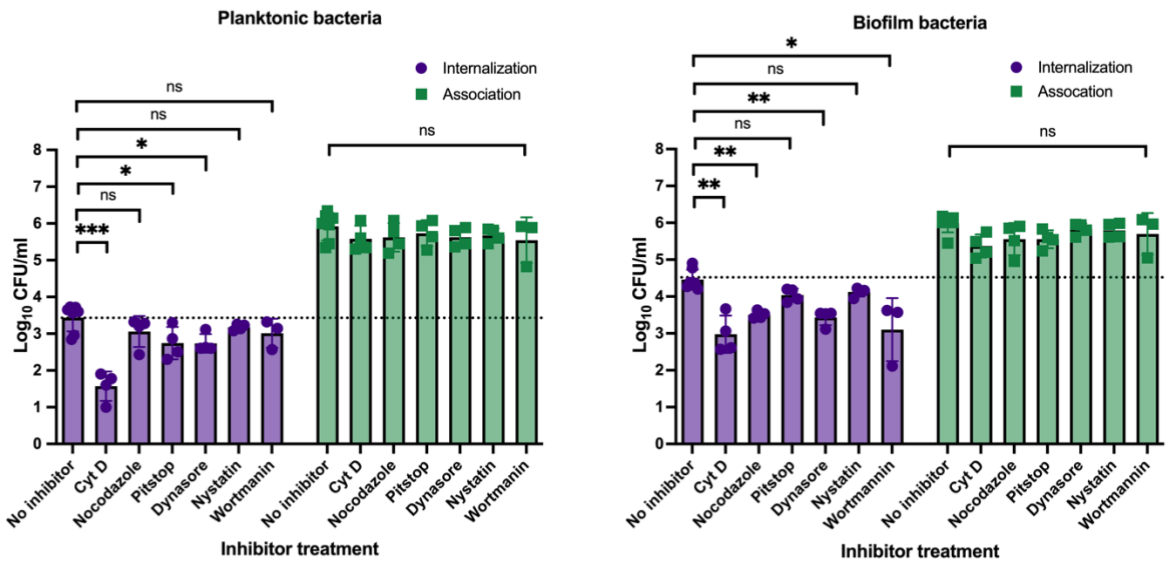

Figure S7. Cellular uptake pathways utilized by intracellular GAS bacteria in respiratory epithelial cells.

To determine the cellular uptake pathways utilized during GAS internalization, live respiratory epithelial (H292) cells were pre-treated with inhibitors targeting proteins involved in uptake pathways, including cytochalasin D (actin, 50 μg/ml), nocodazole (microtubulin, 10 μg/ml), pitstop 2 (clathrin-mediated uptake, 11.8 μg/ml), dynasore (dynamin, 25.7 μg/ml), nystatin (lipid-raft mediated uptake, 7.5 μg/ml) or wortmannin (macropinocytosis, 100 μg/ml) for 1 h. Inhibitor- or non-treated cells (No inhibitor) were inoculated with *S. pyogenes* planktonic (**A**) or biofilm (**B**) GAS-771 in RPMI supplemented with 2% serum. Cells were inoculated with bacteria at 34°C for 4 h without antibiotic treatment, or 2.5 h followed by antibiotic treatment for 1.5 h to eliminate extracellular bacteria. Total bacterial association to the cells (Association, green bars), or internalization levels (Internalization, purple bars) were assessed by determining the Log_{10} CFUs, 4 h post infection. The dotted line represents the internalization level for the no inhibitor control. One-way ANOVA using Dunnett's multiple comparison tests were used to compare the means of inhibitor treated cells as compared to the non-treated cells ± SD (n=3 experiments). For the internalization experiments each individual significance level is displayed in the figure. For the association data all comparisons were non-significant and only one bar is added to represent this fact. For all statistical analyses *, $P < 0.05$, **, $P < 0.01$, ***, $P < 0.001$, and ns, non-significant difference.
